# A Semi-Interpenetrating Network Sorbent of Superior Efficiency for Atmospheric Water Harvesting and Solar-Regenerated Release

**DOI:** 10.1021/acsami.4c02451

**Published:** 2024-05-08

**Authors:** Samar N. Abd Elwadood, Andreia S. F. Farinha, Yasser Al Wahedi, Ali Al Alili, Geert-Jan Witkamp, Ludovic F. Dumée, Georgios N. Karanikolos

**Affiliations:** † Department of Chemical Engineering, Khalifa University, Abu Dhabi 127788, UAE; ‡ Center for Catalysis and Separations (CeCaS), Khalifa University, Abu Dhabi 127788, UAE; § King Abdullah University of Science and Technology (KAUST), Water Desalination and Reuse Center (WDRC), Division of Biological and Environmental Science and Engineering (BESE), Thuwal 23955-6900, Saudi Arabia; ∥ Abu Dhabi Maritime Academy, Abu Dhabi Ports, Abu Dhabi 54477, UAE; ⊥ DEWA R&D Center, Dubai Electricity and Water Authority (DEWA), Dubai 564, UAE; # Center for Membranes and Advanced Water Technology (CMAT), Khalifa University, Abu Dhabi 127788, UAE; ¶ Research and Innovation Center on 2D Nanomaterials, Khalifa University, Arzanah Precinct, Sas Al Nakhl, Abu Dhabi 127788, UAE; ∇ Department of Chemical Engineering, University of Patras, Patras 26504, Greece; ○ Institute of Chemical Engineering Sciences, Foundation for Research and Technology-Hellas (FORTH/ICE-HT), Patras 26504, Greece

**Keywords:** moisture sorption, water harvesting, hygroscopic hydrogels, alginate hybridization, photothermal harvester

## Abstract

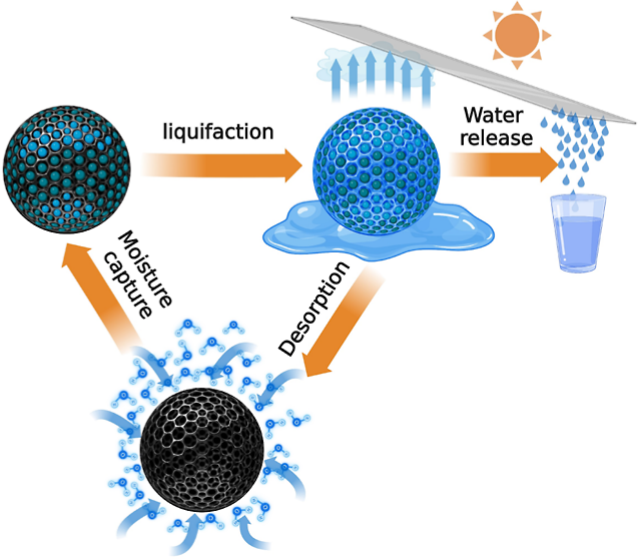

Water is readily available nearly anywhere as vapor. Thus, atmospheric water harvesting (AWH) technologies are seen as a promising solution to support sustainable water production. This work reports a novel semi-interpenetrating network, which integrates poly(pyrrole) doped with a hygroscopic salt and 2D graphene-based nanosheets optimally assembled within an alginate matrix, capable of harvesting water from the atmosphere with a record intake of up to 7.15 g_w_/g_s_. Owing to the incorporated graphene nanosheets, natural sunlight was solely used to enable desorption, achieving an increase of the temperature of the developed network of up to 71 °C within 20 min, resulting in a water yield of 3.36 L/kg_S_ in each cycle with quality well within the World Health Organization standard ranges. Notably, after 30 cycles of sorption and desorption, the composite hydrogel displayed unchanged water uptake and stability. This study demonstrates that atmospheric water vapor as a complementary source of water can be harvested sustainably and effectively at a minimal cost and without external energy input.

## 1 Introduction

Approximately 500 million individuals experience severe water shortages throughout the year, and around 4 billion people reside in water-scarce regions. Desalination and rainwater harvesting are some of the technologies developed to address water scarcity.^1^ Piping freshwater over long distances is a requirement for regions without access to water, but these projects are expensive and unsustainable, leaving 783 million people without reliable access to safe drinking water.^2,3^ Hence, innovative and sustainable technologies to provide clean water without relying solely on either seawater desalination or extraction from aquifers are required to meet the growing demand for water in such regions.

Atmospheric water harvesting (AWH) is an alternative approach to address water scarcity and increase water security through decentralized water production.^4^ Atmospheric water is estimated to be around 13 × 10^15^ m^3^ at any given time, therefore largely dwarfing all available sources of liquid freshwater both in volumes and availability since the majority of liquid freshwater is stored within icecaps.^5^ Dew harvesting, sorbent-assisted methods, and fog capture are among the current low-tech passive AWH technologies that can be adapted to various regional geographical and meteorological conditions and generate deionized water at low footprints and energy requirements. Sorbent-assisted AWH is viewed as the most appealing modular and decentralized approach, especially for locked-land countries with no direct access to ocean shores, required to set up desalination plants.^6^

The optimization of the sorbent material is critical since operational factors, such as regeneration temperature and sorption isotherm types, will significantly impact both performance and industrial upscaling.^7^ Cost-effectiveness, reusability over multiple adsorption/desorption cycles and kinetics of sorption/desorption, as well as eco-friendliness and hydrothermal stability of the materials, are crucial performance criteria to consider upon designing water-sorbent materials.^8,9^ Inorganic adsorbents, such as activated carbon, silica gel, and zeolites with water uptakes of 0.64, 0.4, and 0.1–0.42 g_w_/g_ads_, respectively, were first developed for application as sorbents.^10,11^ However, the equilibrium water uptake is still limited, and materials offering greater porosity and specific surface area, as well as interaction sites with water molecules, were required. Ultraporous aerogels with large specific surface areas on the order of 600 to 1000 m^2^/g, and extreme porosities, between 85 and 99%, offering capture capacities up to 1.4 g_w_/g_s_ were recently found to partly fill these gaps,^12^ while super absorbent gels (SAGs) based on hydrophilic polymers that incorporate hygroscopic salts enclosed in highly porous matrices, led to capacities up to ∼4–5 g_w_/g_s_.^13−15^ Among these, a super moisture absorbent gel (SMAG) stands out, boasting a capacity of up to 6.2 g_w_/g_s_ at 90 RH %. This gel system is composed of a thermoresponsive Poly *N*-isopropylacrylamide (PNIPAM) and polypyrrole, which acts as a trigger for water uptake. Another remarkable gel system is the super hydrophilic calcium alginate hydrogel beads doped with photothermal functionalized carbon nanotubes (CNTs), which exhibits a moisture sorption up to 5.6 g_w_/g_s_ at 90% RH, i.e., being one of the highest reported.^16^ Yet, more work is needed to enhance recyclability, desorption, and water recovery under real-world conditions.

In hybrid polymeric systems, various network architectures can arise, including the double network (DN), the interpenetrating network (IPN), and the semi-interpenetrating network (semi-IPN). In the DN architecture, two independent polymeric networks are present within the hybrid gel without interpenetration. In contrast, in the IPN architecture, the polymer networks interpenetrate one another at the molecular level, and both are cross-linked. In the semi-IPN architecture, one polymer forms a cross-linked 3D network while the other polymer lacks cross-linking, and the two networks are not chemically bonded.^17^ In this study, an optimized hybrid sorbent comprising a semi-IPN was developed to generate an architecture whereby a hydrophilic cross-linked binary alginate (BA) was combined with graphene oxide (GO) as an effective light absorber for solar-enabled evaporation and hygroscopic polypyrrole doped with chlorine (PPyCl). Accordingly, the sorbent’s name “BAGY” is shorthand for the key components of the sorbent, which stands for binary alginate, graphene, and pyrrole, reflecting its synergistic composition. Polypyrrole shows a high density of nitrogen functional groups that can efficiently capture and liquefy moisture from the atmosphere with low-cost and simple production, also exhibiting good biocompatibility.^18^ Besides, the abundance of hydrophilic functional groups in graphene oxide (GO) and sodium alginate’s backbone chain, such as hydroxyl and carboxyl groups, enhance the material’s ability to absorb moisture.^19^

## 2 Experimental Section

### 2.1 Chemicals

Calcium chloride and lithium chloride (∼99% anhydrous) were obtained from Sigma-Aldrich. Sodium Alginate (SA, ≥99% with M/G = 1:2, 1:1, and 2:1) was obtained from Shanghai Macklin Biochemical Co., Ltd. The following chemicals were used for graphene oxide (GO) preparation: 99.9% metals basis–10 mesh natural graphite flakes from Alfa Aesar (Germany), potassium permanganate (99% KMnO_4_) from Fisher Scientific (USA), hydrogen peroxide (35% H_2_O_2_), and various acids, namely, sulfuric acid (97% H_2_SO_4_), phosphoric acid (85% H_3_PO_4_), and hydrochloric acid (37% HCl) from Merck (Germany). Ammonium persulfate, pyrrole, lithium chloride, and HCl were acquired from Sigma-Aldrich and used for the preparation of polypyrrole doped with chlorine (PPyCl).

### 2.2 BAGY Synthesis

#### 2.2.1 Preparation of Polypyrrole Doped with Chlorine

A 2.29 wt % solution of ammonium persulfate (APS) was prepared and labeled as (A). A 6.47 wt % solution of pyrrole was sonicated for 20 min and labeled as (B). In 50 mL of 1.5 M HCl aqueous solution, 2.1 g of lithium chloride was dissolved. A and B were alternately and dropwise introduced to the LiCl solution under stirring. Polymerization was performed over 5 min, followed by washing and filtering with DI water 4 times to remove any excess acid or APS.

#### 2.2.2 Composite Sorbent Preparation

To prepare binary cross-linked alginate (BA), sodium alginate was slowly added to water under stirring at 80 °C in order to ensure that sodium alginate was completely dissolved. In order to prepare the binary alginate with GO composite (BAG), 5% wt, the synthesized GO, using the modified Tour’s approach,^20−22^ was suspended in water and mixed under stirring for 2 h followed by ultrasonication for 20 min using a SB 5200 Scientz ultrasonicator. To prepare alginate composite with GO and PPyCl composite (BAGY), PPyCl powder and 5 wt % GO were first suspended in water.

**Figure 1:**
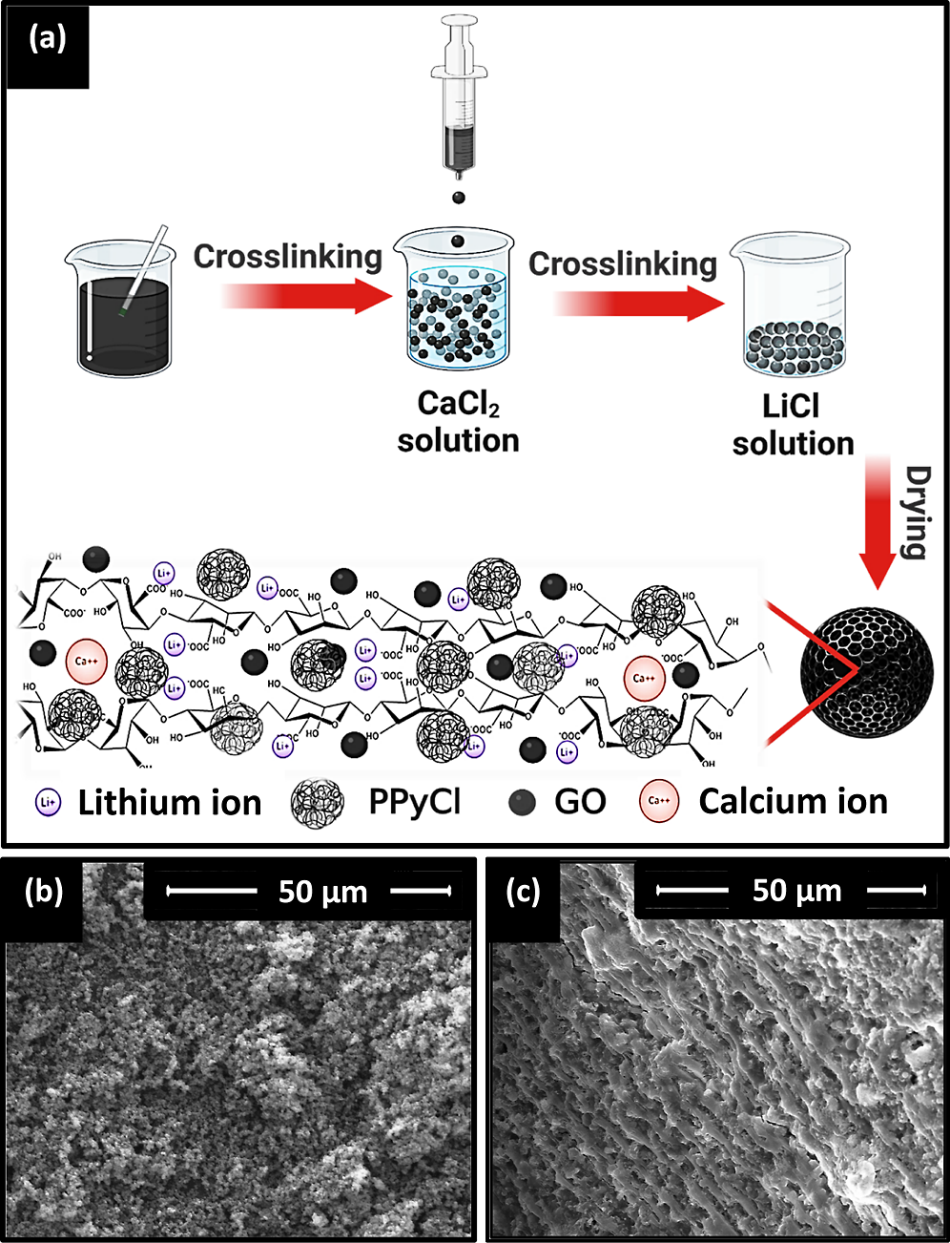
(a) Schematic of step-by-step preparation of BAGY via gelation of alginate, GO, and PPyCl by saturated CaCl_2_ and LiCl solutions, including a schematic of the inner structure of the obtained BAGY, SEM image of (b) the morphology of PPyCl’s surface, and (c) the inner structure of the synthesized BAGY.

Then, sodium alginate was slowly added to these suspensions under magnetic stirring at 80 °C until complete alginate dissolution, after which the as-prepared solutions were dropped into a saturated calcium chloride solution. After 12 h, the formed calcium alginate particles were filtered and soaked in a sodium chloride saturated solution. After 24 h, the formed beads were separated and washed several times with ethanol (Figure S1a) and dried overnight at 110 °C (Figures 1a and S1b).

### 2.3 Characterization

Scanning electron microscopy (SEM) images were obtained using an FEI Quanta 250 FEG instrument with energy dispersive X-ray spectroscopy (EDS) capability at 10 kV and an operating distance of 12 mm. Prior to SEM analysis, the samples were placed on aluminum stubs using adhesive tape and coated with gold, while they were also subjected to EDS examination at an operating length of 12 mm and a voltage of 20 kV. A Micromeritics 3Flex adsorption analyzer (Micromeritics Instruments Corporation, USA) was used to evaluate the textural characteristics of the adsorbents and determine their surface area and porosity using ultrapure nitrogen gas (99.99%) at 77 K with equilibration interval of 10 s in relative pressure *P*/*P*_0_ range from 0 to 0.99. The Brunauer–Emmett–Teller (BET) model was used to determine the surface area. Sorption measurements were performed after the samples were degassed overnight at 80 °C under vacuum. Fourier-transform infrared spectroscopy (FTIR) of the developed composites was carried out using a Bruker Vertex 70 FTIR spectrophotometer. Sixty-four scans were gathered at a resolution of 4 cm^–1^, covering the wavelength range 4000–400 cm^–1^, while baseline correction and background acquisition were performed. X-ray diffraction (XRD) analysis was performed by using a Bruker D2 Phaser. A holder with zero-background was used to gather the XRD patterns across a 2θ range of 4 to 70° with a step size of 0.02. X-ray photoelectron spectroscopy was carried out on a Thermo Fisher Scientific, ESCALAB 250Xi XPS spectrometer. With a resolution of 0.050 eV and a passage power of 20 eV, XPS spectra were collected. A Goniometer Contact angle analyzer (DSA25, Kruss) was used to determine the water surface contact angle (WCA) of the samples at ambient temperature. The WCA was calculated over time with the use of instrument software. Raman spectra were obtained under ambient conditions using an alpha300 RA Raman-AFM combination in the 100–3600 cm^–1^ Raman shift range, following calibration with a Si wafer at 520.7 cm^–1^. Rheological properties were performed with an AR 2000EX rheometer by a TA Instruments in frequency sweep mode. An Instron mechanical tester was used to perform mechanical compression tests on the composites. Thermogravimetric analysis (TGA) was carried out at temperatures between 35 and 400 °C and a heating rate of 10 °C/min under N_2_ using a STA 449 F3 Jupiter, NETZSCH TGA analyzer. Differential scanning calorimetry (DSC) could detect phase change using the STA 449 F3 Jupiter, NETZSCH analyzer coupled to a modular humidity generator (MHG) under a nitrogen purge of 20 mL/min. The solar irradiation for the indoor and photothermal conversion experiments was supplied by a solar simulator (Sol3A 94123A) with a 1600 W xenon source lamp and 12 in. × 12 in. area with an Air Mass 1.5G filter to make up a standard 1 Sun illumination. The temperature of the sample surface was measured by an IR camera (FLIR). Light absorbance of the composites was quantified by using ultraviolet–visible–near-infrared spectroscopy (UV–vis–NIR) on a Shimadzu (UV-2600) UV–vis spectrophotometer. Measurements were obtained between 200 and 1400 nm. The climate data were collected by a Davis vantage pro sensor. The recovered water purity was characterized using inductively coupled plasma-optical emission spectrometry (ICP-OES) 5100 by Agilent Technologies.

### 2.4 Water Vapor Adsorption Studies

#### 2.4.1 Water Capture Experiments

##### 2.4.1.1 Static Tests

The kinetics of samples were investigated using static sorption at constant temperature and RH. The pan containing the sample was brought into the testing chamber in order to acquire information on mass change over time at three RHs values (10, 40, and 70% RH) and with the temperature set to a predefined value (23 °C).

##### 2.4.1.2 Dynamic Tests

The instrument used for the dynamic tests was a vapor sorption analyzer (Q5000SA) from TA Technologies. Deliquescent salts were initially utilized to test or calibrate moisture generators, in line with ASTM E 2551. The relative humidity was varied from 5 to 98%, and the deliquescence points of the salts were determined gravimetrically at the set temperatures.

The steps listed below were used to obtain isotherms at various temperatures: The temperature in the moisture compartment was regulated to 60 °C, and a sample pan was introduced. RH in the humidity chamber was set to 0%, and samples were equilibrated for 60 min or until their weight variation was less than 0.05%. Then, the temperature was set to 23 °C, and the RH was changed by a 10% RH step. The sample weight change was equilibrated, and the RH was raised by an additional interval of 10% each time. In order to obtain the desorption branches of the isotherms, the preceding steps were repeated in opposite order (from higher to lower RHs). The same procedure was carried out at each temperature at which isotherms were obtained.

##### 2.4.1.3 Water Capture Optimization

The effect of altering the relative concentrations of the PPyCl and sodium alginate (SA) components was studied in order to maximize the water uptake of the composite. Samples are prepared with different PPyCl/SA weight ratios in the range between 0 and 200 wt % with intervals of 10%. The performance of the samples was evaluated and compared under low, moderate, and high humidity levels in a lab-made humidity box (Figure S2) at room temperature and RH of 40, 70, and 90%.

##### 2.4.1.4 Indoor Testing

The dried samples loaded in a Petri dish were tested in laboratory conditions using a clear box. The box upper transparent cover was left exposed to allow ambient moisture to be captured while the light was blocked from the generator. A microbalance monitored the sample weight initially until an equilibrium weight was attained. By tracking the weight change of the samples over time, we could obtain the water uptake profile. After being exposed to ambient humidity for 48 h, at conditions of 23–25 °C and 60–80 RH %, the materials collected the maximum amount of moisture (saturation) from the air.

##### 2.4.1.5 Outdoor Testing

At the location KAUST University campus in Thuwal, Saudi Arabia, the dried samples were placed in a transparent box and exposed to the atmosphere to collect moisture from 6:00 pm on September 13, 2022, until 7:00 a.m. on September 14, 2022.

#### 2.4.2 Water Release Experiments

##### 2.4.2.1 Desorption Behavior

The desorption behavior was evaluated under a N_2_ environment. The instrument used was the Q5000SA vapor sorption analyzer. Also, desorption was investigated under atmospheric air in the humidity box. The sample desorption behavior over time was examined at three temperatures (60, 70, and 80 °C) and 70% RH.

##### 2.4.2.2 Indoor Measurement of Desorption

Indoor laboratory conditions with a source of artificial sunlight were applied to investigate the composite’s potential to accelerate moisture desorption while being exposed to sunlight of 1 sun intensity, and both mass and temperature variations were recorded over time. Enclosing the sample container within a transparent, airtight cover during desorption allows hot water vapor to be desorbed from the sorbent and condense on the colder inner walls. For condensation, no cooling system was employed. The box’s bottom was isolated to prevent heat loss through it, and the sides of the container were insulated using sheets of high reflectivity and low thermal conductivity to keep their temperature comparatively lower than the surroundings.

##### 2.4.2.3 Outdoor Measurement of Desorption

The water release effectiveness of the samples was assessed at the KAUST campus in Thuwal, Saudi Arabia, from 7:00 am on September 14, 2022, to 6:00 pm on September 14, 2022, using natural solar radiation in outdoor environment conditions. The apparatus was covered at 7:00 am to start the desorption and precipitation process. Temperature changes, fluctuations in sun radiation, and changes in the sample mass were monitored.

#### 2.4.3 Cyclic Testing

The regenerability and reusability of the developed BAGY were examined throughout several sorption/desorption cycles. The beads were initially dried at 80 °C for 10 h. For the first cycle (activation cycle), the dried sample was exposed to moisture at a certain relative humidity (70% RH) until equilibration was performed to make sure it was thoroughly hydrated and activated. The hydrated composite was then heated in order to release the sorbed water at 80 °C. The desorption conditions for the subsequent cycles were the same as those in the first cycle, and sorption was carried out at 70% RH for an optimized duration.

### 2.5 Water Sorption Isotherm Models

Nonlinear regression was used to fit the acquired experimental sorption isotherms to various models, namely, GAB, Langmuir, FHH, and Freundlich. The formulas and the model’s parameters are presented in refs (23 and 24).

### 2.6 Water Vapor Sorption Kinetics

Pseudo-first-order and pseudo-second-order models were used to study the sorption kinetics of the samples.^25,26^

## 3 Results and Discussion

### 3.1 Characterization of the Hybrid Sorbents

BAGY was prepared by cross-linking alginate chains around PPyCl clusters. The final composite was obtained as beads with a diameter of 2–3 mm. PPyCl showed a globular submicrometer structure and a granular amorphous morphology (Figure 1b), while BAGY showed a dense, porous, interconnected, and layered fine network with irregularly sized macro pores (Figure 1c), providing channels for sufficient water diffusion and vapor escape during regeneration. The presence of large pores was also confirmed from N_2_ adsorption isotherms of BAGY at 77 K, which exhibited high uptake of relative pressure (*P*/*P*_0_) values higher than 0.8 (Figure S4).^18,27^

**Figure 2:**
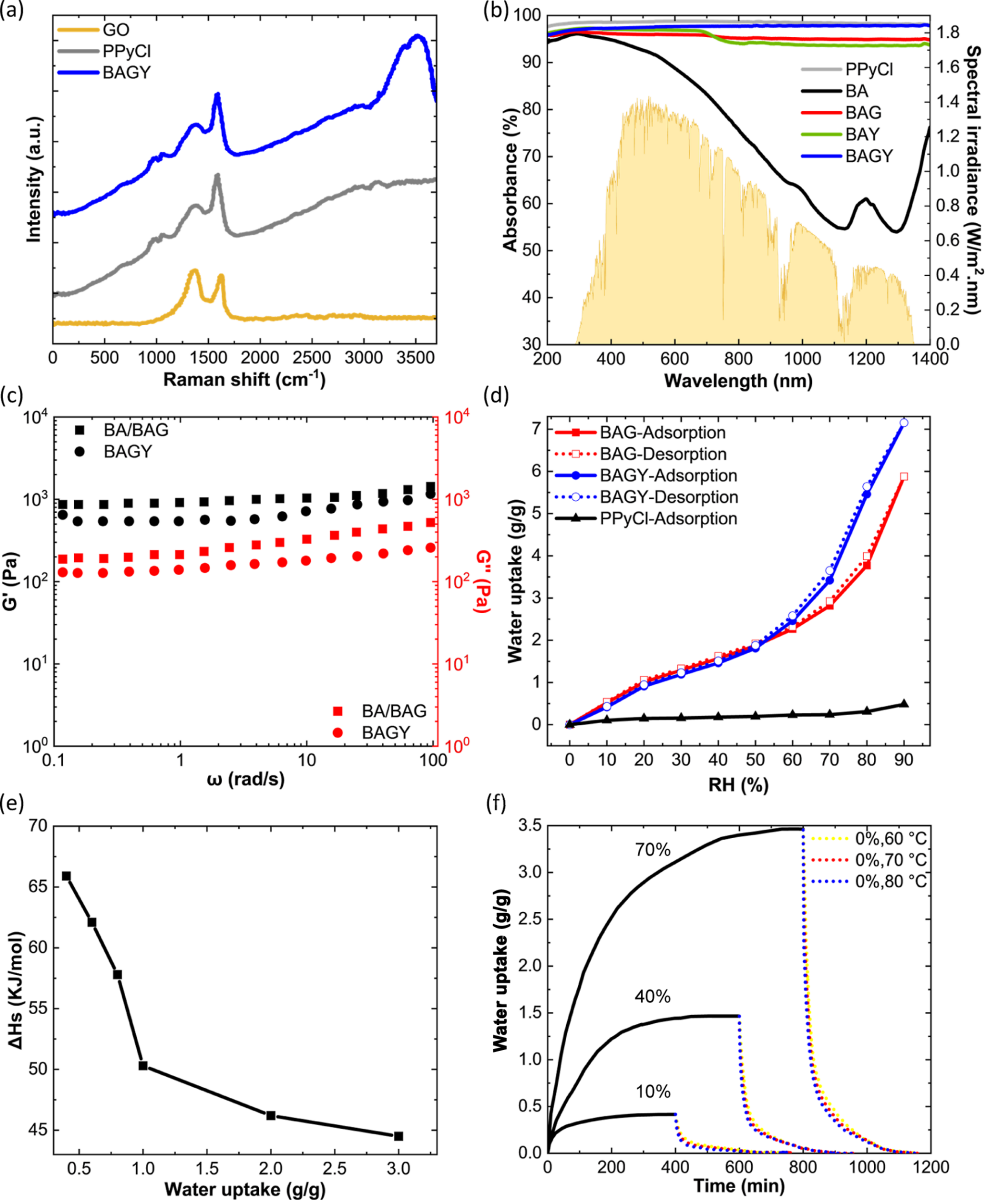
(a) Raman spectra of GO, PPyCl, and BAGY, (b) UV–vis–NIR absorption spectra of PPyCl and alginate cross-linked composites, (c) storage and loss modulus of composites, (d) water vapor sorption isotherms of PPyCl, BAG, and BAGY at 23 °C, (e) isosteric heat of water vapor sorption on BAGY, and (f) water uptake kinetics for BAGY at 10, 40, and 70% RH under N_2_ environment at 23 °C and water release kinetics at 0% RH and 60, 70, and 80 °C.

The instantaneous permeation of water droplets into BAGY, indicated by a 0° contact angle, underscores its exceptional hydrophilicity, a result of the combined effects of alginate and the integrative interaction between graphene oxide (GO), sodium alginate (SA), and water via hydrogen bonding. Further insights into the material’s composition and the bonding of water to its structure were gleaned from Raman spectroscopy (Figure 2a). This revealed the integration of PPyCl and GO within the alginate matrix, marked by similar spectral patterns for PPyCl in the composite and distinct hydrogen bond vibrations. These findings show that PPyCl and GO are not simply blended or mixed but that GO is entrapped within PPyCl clusters and alginate chains and there is strong interaction between PPyCl and GO, providing evidence of the intricate hybrid structure of BAGY, contributing to its functionality.

In evaluating our materials’ photothermal conversion efficiency, a simulated sunlight source revealed significant temperature increases within the first 10 min, with BAGY demonstrating a remarkable ability to sustain a higher equilibrium temperature (71 °C), indicative of its superior solar energy utilization. Consequently, it is imperative to highlight that BAGY obtained above 98% solar conversion efficiency in UV–vis–NIR tests (Figures 2b and S13). Yet, the light absorption was significantly lower in the case of BA (down to 54%), suggesting that the synergetic effect of alginate with the PPyCl and GO greatly enhanced the light absorption.^28^

The BAGY’s mechanical attributes were also assessed under compression (Figure S9). The compressive stresses for the wet BAGY (0.5 MPa) were lower by ∼12 times compared with the dried sample (6.4 MPa). In the stress–strain curves, BAGY displayed an elastic linear domain, suggesting a typical elastomeric behavior. After absorbing water vapor from the air, the storage modulus *G*′ was found to be much higher than the loss modulus *G*″ (Figure 2c). The low *G*″ value of BAGY also reveals a significant restriction of the slippage between polymer chains, suggesting that the PPyCl clusters were inserted into the alginate chains, creating an interpenetrating hybrid structure.^29^ To provide a comprehensive overview of these findings, detailed discussions of FTIR, XRD, XPS, EDS, TGA, and DSC characterizations have been included in Sections S1.3–S1.11 of the Supporting Information.

### 3.2 Water Vapor Sorption Evaluation

The water uptake was maximized by adjusting the content of PPyCl, sodium alginate (SA), and gelation salt (Section S1.12), resulting in the identification of an optimized sample (BAGY) with a PPyCl to SA ratio of 200 wt %. This optimized sample was utilized in all subsequent tests, encompassing dynamic, static, indoor, outdoor, cyclability, and desorption tests.

#### 3.2.1 Sorption Isotherms and Mechanism

Dynamic sorption studies at various RHs and temperatures were used to derive water sorption isotherms gravimetrically. The dried samples were examined at 23 °C and RH levels between 10 and 90%, as shown in Figure 2d. Both the BAG and BAGY samples showed similar behavior, indicating an analogous process for water vapor sorption across both materials, which suggested that PPyCl did not significantly impact the isotherm type of BAGY. However, when compared with BAG, the presence of PPyCl enabled synergistic effects that enhanced the equilibrium water sorption by 22 wt % (7.15 g_w_/g_BAGY_ at 90% RH) (Figure 2d). BAGY’s equilibrium water uptake decreased by 42 wt % with temperature increase between 23 and 53 °C (Figure S18), indicating that the water uptake process is exothermic.

The effectiveness of BAGY was evaluated in terms of the variance in water release and uptake during a sorption–desorption cycle at 23 °C, and no hysteresis was found during these tests, suggesting that complete water desorption at all RHs may be achieved. The water vapor sorption on BAGY at different temperatures was fitted to four isotherm models, i.e., Langmuir (Figure S19a), Freundlich (Figure S19b), Frenkel-Halsey-Hill (FHH) (Figure S19c), and Guggenheim–Anderson–de Boer (GAB) (Figure S19d). Table S3 illustrates the parameters derived by using the aforementioned models. The FHH and GAB were found to provide the best fit for the sorption isotherms for BAGY based on the highest correlation coefficient values, which agrees with similar polymeric-based gels in literature, such as *N*-isopropylacrylamide and acrylamide-based gel.^23,30,31^ The BAGY samples exhibited a type II sorption isotherm without showing a saturation point (Figure 2d), suggesting the existence of a large density of large pores within the materials, as confirmed by SEM images and liquid N_2_ adsorption isotherms. The accompanying moisture-capture process is more complex than that predicted by existing models and theories. Water sorption and diffusion in composites are governed by several processes, including salt-vapor chemical reactions, the formation of hydrogen bonds between water and polymeric anions backbone groups, and general absorption within the network leading to polymer swelling.^32^ Water transits in hydrogels occur via a multistep process whereby water vapor is first caught and dispersed to the outer pore space in the first stage. While the second phase includes water molecules moving to the interior pores space. In the final stage, water molecules diffuse into the porous internal structure via capillary condensation and liquefaction. Consequently, according to Smith’s hypothesis, the sample isotherm may be partitioned into two zones (Figure 2d).^33^ The first part entails condensing water molecules on the network’s surface (corresponding to the convex part of curve between 0 and 40% RH), which is primarily governed by relative humidity. During the second part, water molecules attach to the polymeric sorbent’s interior structure, resulting in a concave curvature (above 50% RH).^33^ Since water vapor could not efficiently diffuse into the interior porous structure with RH of less than 50%, the water vapor sorption increased gradually with RH rise, leading to a water uptake up to 1.8 g_w_/g_BAGY_ at 23 °C at 50% RH. Nonetheless, capillarity can act as a driving force for rapid water transport since the hydrophilic gel matrix is strongly interconnected. The water vapor sorption capacity rose much more abruptly in the second zone with RHs between 50 and 90%. Indeed, the water uptake reached almost 7.15 g_w_/g_BAGY_ at 23 °C at 90% RH while the majority of the water was sorbed as bulk liquid water. This rise might also be due to the molecular level interactions between PPyCl and alginate, which facilitates internal water rearrangement and activates the sorption sites to harvest additional water molecules due to their high polarity, while the NH^+^/Cl^–^ charge pairs of polypyrrole can further boost the hydrogel’s ability to sorb water.

The water uptake capacity of PPyCl, on the contrary, has a weaker reliance on the RH change (Figure 2d). However, a molecular dynamics optimized model of PPyCl has demonstrated effective trapping of water molecules by the PPyCl solvation action.^34^ Despite its effective moisture capture, PPyCl’s water uptake is still insufficient to fulfill the AWH demand. The superhydrophilicity of alginate may transcend such constraints as a result of utilizing both M and G blocks in moisture capture instead of activating only one of them in the case of alginates cross-linked with mono salts. It also has strong coordination bonds, ionic bonds, and polar functional groups, all of which are necessary for binding polar water molecules. These networks feature mobile ion pairs made up of polymer electrolyte ions, as per the theory of network structure.^16^ Before the hydrogel absorbs water, its long, cross-linked polymer chains have formed a nonionized solid bundle. When a polymer absorbs moisture, its hydrophilic group becomes hydrated, which causes different ion concentrations inside and outside the network.^16^ Then, the osmotic pressure generated inside and outside of the structure motivates moisture absorption inside the structure.

#### 3.2.2 Isosteric Heat of Sorption

The desorption enthalpy is governed by the status of the sorbed water in BAGY, in which the majority of water is typically present as liquid water, while the remaining water is bound to the polymer’s structure.^35^ The presence of these different water states inside the hydrated hydrogel has a number of important consequences, including the value of enthalpy of vaporization.^17^ The Clausius–Clapeyron equation was used to fit the experimental data from BAGY and determine the isosteric heat of sorption. Figure 2e shows isosteric heat of sorption (Δ*H*_s_) at various sorption capacities, while values of Δ*H*_s_ together with correlation coefficients are displayed in Table S4. It should be noted that the resolution and interpolation applied to the sorption isotherms typically affect this approach’s performance,^36^ making calorimetric approach produce more precise results. The enthalpies of desorption for BAGY using DSC are shown in Table S5. Since water molecules are bonded to the BAGY’s surface functional groups at lower RH values, which commonly manifested as bounded water, the Δ*H*_s_ were comparatively higher at lower sorption capacities. Despite this, when the RH increased, water gradually penetrated deeper into the sorbent’s structure, and most of the water existed as bulk liquid and weakly bonded molecules; thus, the Δ*H*_s_ values decreased and approached the values for the enthalpies of evaporation of pure water.

#### 3.2.3 Sorption Kinetics

The sorption kinetics is one of the variables that affects the selection criteria for sorbents for AWH. Sorption/desorption kinetic behavior of BAGY is examined in a nitrogen atmosphere and under realistic simulated conditions. Water uptake versus time is depicted in Figures 2f and S19a. At 70% RH under N_2_ atmosphere, the rate of water sorption by BAGY in the first 60 min is 0.02 g/g·min, which is 62.4% of its equilibrium water uptake (3.42 g/g). Under air, the rate of water sorption (0.02 g/g·min) achieves 33% of its saturation water uptake in the first 60 min. The BAGY’ water sorption equilibrium under N_2_ was reached in 200 min at 10% RH and in 360 min at 40% RH, while at 70% RH, equilibrium was reached in 650 min (see Figure 2f).

However, BAGY reached equilibrium under air in 290 min at 10% RH and 475 min at 40% RH, whereas it took 1200 min at 70% RH (Figure S22a). The variations in equilibrium times may be attributed to increased composite sensitivity to humidity with successive RH increases, requiring longer time to reach equilibrium at higher RHs. As the sorption process started, there was a fast rise in water intake under both N_2_ and an air atmosphere. Chemisorption, capillary condensation, and intermolecular interaction between the numerous sorption sites in the composite and water vapor molecules contributed to the rapid sorption. Moreover, through the insertion of PPyCl and GO in the alginate network, the water sorption kinetics of BAGY was enhanced by creating a porous interpenetrating structure overcoming the sluggish diffusion via tightly packed alginate chains. Then, the sorption rate decreased, indicating that water molecules occupied the voids inside the gel polymer structure, making it harder for new molecules to penetrate. Fittings of experimental data with the pseudo-first-order and pseudo-second-order kinetic models are shown in Figure S22b, while relevant fitting parameters are listed in Table S6. For the pseudo-first-order and pseudo-second-order kinetic models, the correlation coefficients are higher than 0.9. However, the pseudo-second-order model accurately captures the behavior of BAGY with a correlation coefficient of 0.99, and it is able to describe water sorption through both physical and chemical sorption.

Our investigation has revealed that the physical form of the sorbent, whether it be powder, beads, or rods, plays a role in the rate of water sorption. This investigation deepens our understanding of BAGY’s capabilities by incorporating kinetic data, not only underscoring its consistent sorption capacity across different forms but also highlighting the varied rates at which equilibrium is achieved. The powder form of BAGY was found to reach equilibrium 8.6% faster than the beads, with the beads outpacing the rods by 3.2%. This indicates that the powder’s increased exposed surface area significantly enhances water vapor interaction, thereby accelerating sorption. Choosing beads for the development of BAGY was a deliberate decision intended to balance material consistency, structural integrity, and practicality in real-world AWH scenarios. Beads were chosen for their ease of handling and compatibility with existing water capture systems, rendering them a suitable choice for practical application.

The BAGY desorption rate was noticeably greater than the sorption rate because solar heat was employed as a trigger. Figures 2f and S19a show the sample weight change over time at 60, 70, and 80 °C. A dependence between the temperature and the rate of water release is demonstrated. The sample weight at the end of the desorption test was not substantially different among the various desorption temperatures tested, even though the water desorption rate was higher at higher temperatures (80 °C). Therefore, desorption at lower temperatures (60 °C) is preferred to minimize energy need, despite the fact that the water evaporation rate was lower. Such temperatures are readily attained with solar radiation since the temperature of dry BAGY can reach 70 °C in 20 min under 1 sun of irradiance (Figure S13), suggesting that water recovery can be achieved in real-condition experiments under solar radiation.

### 3.3 Water Collection Experiments

In this section, both indoor and outdoor experiments were conducted to assess the potential of using BAGY for real-world AWH. The tests first included standardized calibration and optimization in indoor conditions prior to testing the materials outdoors in realistic settings.

**Figure 3:**
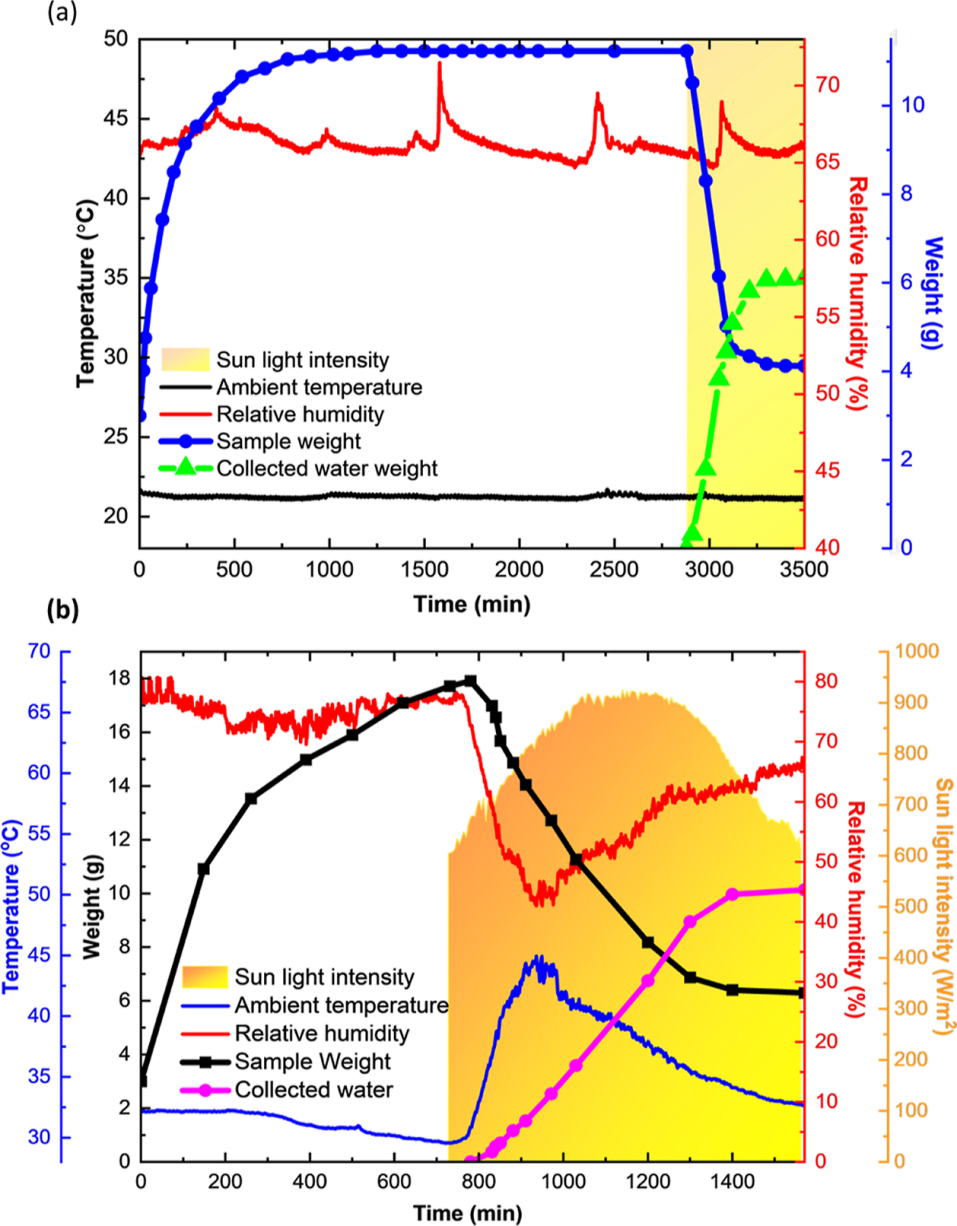
(a) Indoor water harvesting experiment based on a lab-made prototype showing the weight change of BAGY and collected water from experiment and ambient conditions, (b) the weight change of sample and collected water from outdoor experiment and ambient conditions.

Indoor tests were performed to assess BAGY’s potential for practical use at ∼65–70% RH and 21–22 °C. The effectiveness of water release from the hydrogels was investigated in the lab using a source that simulates sunlight and generates around 1 sun intensity. When the sorption phase was complete, the amount of sorbed water was measured to be 2.74 g_w_/g_s_ (Figure 3a). A total of 2.03 g_w_/g_s_ was recovered, resulting in a total water recovery efficiency of 74%, which represents the percentage of the total absorbed water that was successfully released and recovered. The comprehensive indoor testing results leading to the optimization of the materials are presented in Section S1.19.

Additionally, testing was carried out outdoors in the open air and under direct sunlight. From September 13, 2022, at 6:00 pm until September 14, 2022 at 7:00 am, the uncovered box containing the dried hydrogel was exposed to atmospheric water vapor outdoors at the KAUST Campus in Thuwal, Saudi Arabia. Figure 3b depicts the meteorological conditions at the time. The findings show a sample mass gain of 4.8 g_w_/g_s_ during the night time. After sunrise (from 6:00 am), the outdoor temperature rose to about 31 °C while the RH decreased to about 77%. Water sorption continued because the sample had been able to continue capturing moisture since it was not being able to sorb moisture up to saturation overnight. The acquired data showed that after being exposed to air, the composite’s surface temperature increased from 32 to 35 °C (Figure S24b). The heat of sorption, which is produced when the water is trapped, is responsible for the temperature increase. When not adequately controlled or consumed in polymer dissolution, this energy slowly leaks out into the environment, and no further variation in temperature is monitored (Section S1.15).

At 7:00 a.m., the unit was sealed to start the desorption and condensation process; this was done outdoors with no artificial lighting. The sample within the sealed container released water vapor upon exposure to solar light, condensing water on the box walls. Within the first 2 h of sunlight exposure, the hydrogel’s temperature rose as high as 57 °C, while after 6 h it reached its peak temperature of about 69 °C. Between 7:00 am and 6:00 pm, the unit generated 10.1 g of water in total for 3 g of BAGY with a recovery efficiency of 67% with respect to the water sorbed. Several factors contribute to a low water collection efficiency low. One is that some leftover water was attached to the device’s sidewalls, while the second one is that a minor amount of water remained in the composite. Furthermore, because the temperature within the box was slightly greater than the outside temperature, a small quantity of water may have been retained in the inner air. Finally, the moisture lost when the cover was opened and shut contributed to the observed decrease in water collecting efficiency. According to the aforementioned reasons, setup design adjustments are still required. However, it was clear that solar irradiation can produce quick water discharge by absorbing 58% of the solar energy (see Section S1.16). In addition, many AWH systems separate the photothermal component from the sorbent, i.e., heat generation takes place on the photothermal layer and then transmitted to the sorbent, thus resulting in significant heat losses. However, in BAGY, heat generation takes place locally due to the interpenetrating network of GO, PPyCl, and cross-linked alginate. As a result, there is minimal heat loss and a quick increase in temperature.

**Figure 4:**
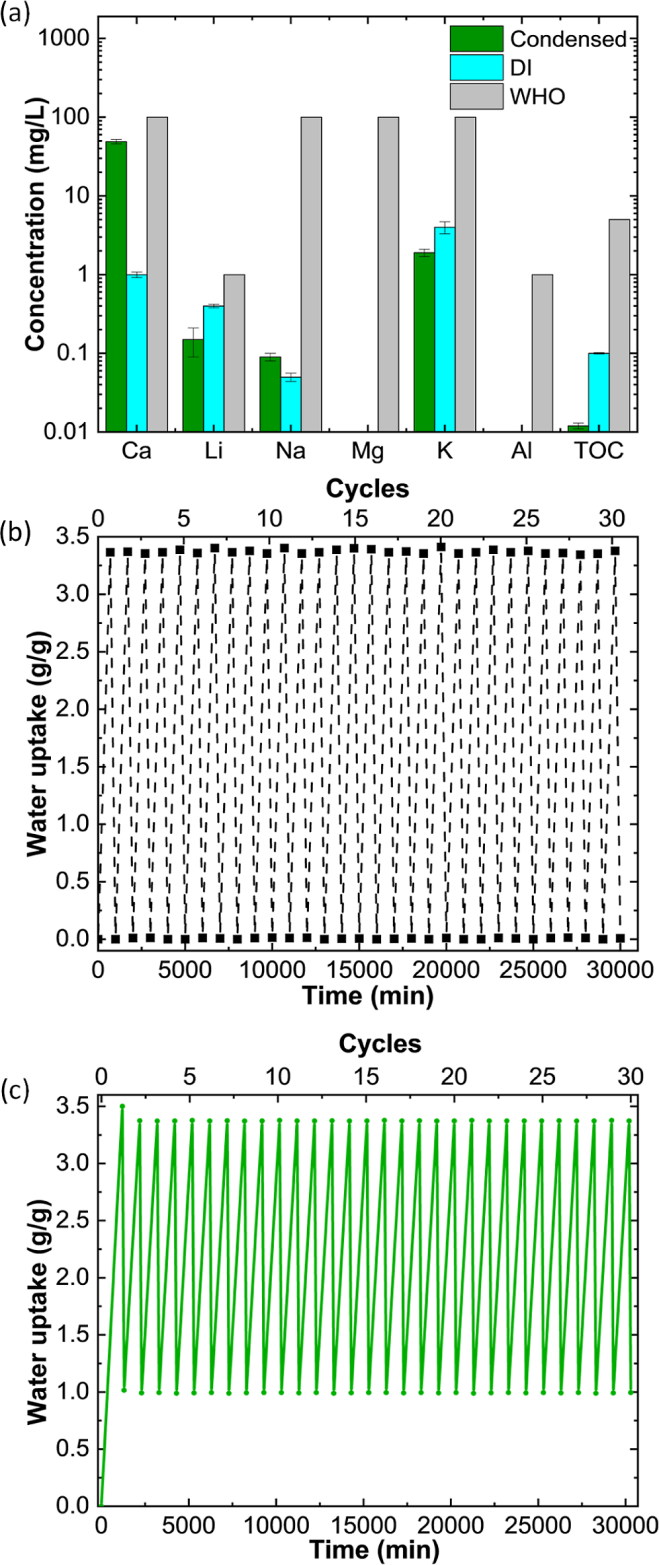
(a) Quality of water collected from the outdoor tests using ICP-OES and TOC analyses compared with deionized water (DI) and World Health Organization (WHO), (b) cyclability upon water sorbing-regeneration of BAGY, and (c) cyclic stability of BAGY under atmospheric air.

Alginates have no major health restrictions, and they are widely used in the food sector.^37^ However, under extreme sorption application conditions, there is a chance that ions may drift and be transferred to the condensation walls with steam. By examining the quality of the liquid water that formed around the sorbent’s beads (Figure S25a) and of the condensed water (Figure 4a) from the outdoor experiment, this study estimated the potential leakage of the C, Ca, Li, Na, and Al ions from the BAGY and assessed it using ICP-OES and Total Organic Carbon (TOC) analyses. The water generated by condensation in the current study has 49 ppm calcium content, making it soft water. Additionally, the water collected had a very low TOC of 0.012 ppm. The observed TOC may be attributable to the potential adsorption of organic carbon from the air. The quantitative characterization of the obtained water shows that the concentration of ions meets the World Health Organization (WHO) standard, indicating the viability and safety of producing clean, fresh water from the atmosphere using the developed system. In addition, Figure S25b displays the fog-capturing behavior. It is apparent from a comparison of the rates at which water is sorbed from fog and moist air (RH = 90%) that water is sorbed from fog more quickly than moisture. These results show that the BAGY can function at low humidity levels and effectively catch water from fog, thus, achieving all-weather atmospheric water harvesting.

Any water harvester should have not only a high sorption capacity but also cyclability. To test this, BAGY was put through 30 successive sorption tests in the laboratory environment, each lasting 700 min at 70% relative humidity, followed by desorption at 0% RH for 300 min at 23 °C. Figure 4b shows that the composite can be completely regenerated after 30 cycles without any discernible capacity drop. The cyclic stability under atmospheric air at 23 °C in terms of water uptake was also examined. A single layer configuration was used to evaluate the composite’s recyclability under natural conditions for 30 continuous sorption/desorption cycles. The dehydrated sample was first kept at a RH of 70% for 1200 min in order to allow water uptake to approach equilibrium as an activation cycle followed by heating for 100 min in an oven at 80 °C. Subsequently, sorption was conducted for 900 min in each cycle. After 30 cycles, no deterioration was found (Figure 4c). Residual free salts could potentially undergo deliquescence under hydration conditions causing clogging of the composite’s porous structure, or they could leach from the polymer matrix. However, this did not seem to have occurred as the water uptake was stable over all cycles tested. Moreover, the experiment indicated that BAGY can be regenerated at temperatures considerably lower than those of standard solid desiccant materials.

**Figure 5:**
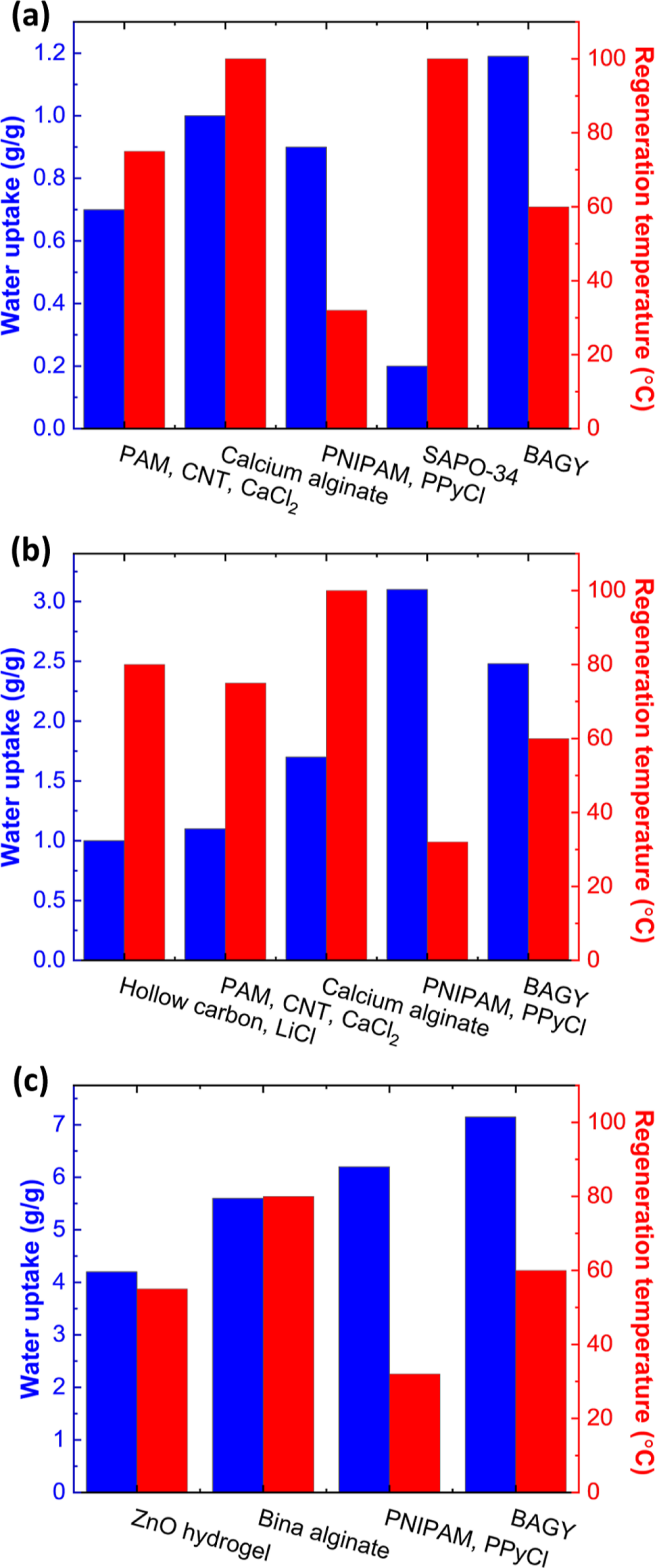
Comparison of top-performing AWH materials based on water uptake and desorption temperature at (a) 30% RH, (b) 60% RH, and (c) 90% RH.

Compared with most AWH and commercial hygroscopic materials, BAGY offers several benefits. The performance of BAGY was evaluated with respect to that of the top performing sorbents in order to determine its competitiveness at regions of low (Figure 5a), moderate (Figure 5b), and high humidity levels (Figure 5c) for AWH. In terms of equilibrium capacity, the BAGY developed in this work has demonstrated the highest sorption capacity (up to 7.15 g_w_/g_s_ at 90 RH %) and a low desorption temperature of 60 °C, resulting in a superior performance with respect to the above two key performance indicators combined and a significantly reduced energy consumption. For instance, at 30% RH, BAGY exhibited a water uptake of 1.19 g_w_/g_s_, while conventional sorbents in arid conditions exhibit lower capacities, such as calcium alginate (1 g_w_/g_s_), SMAG (0.9 g_w_/g_s_), and SAPO-34 (0.2 g_w_/g_s_). At 90% RH, BAGY showed the highest water uptake compared with the top performing sorbents such as SMAG (6.2 g_w_/g_s_) and ZnO gel (4.2 g_w_/g_s_) (Figure 5).

The BAGY composite’s high-water uptake is primarily attributed to its tailored microstructure. The inherent hygroscopic nature of sodium alginate, coupled with the composite’s structural configuration, promotes efficient water molecule entrapment. The low regeneration temperature of BAGY observed at a modest 60 °C can be ascribed to the strategic inclusion of graphene oxide (GO). GO serves as an effective photothermal and heat transfer agent, harnessing solar irradiation and dissipating the thermal energy within the sorbent’s matrix to expedite water molecule release. This photothermally enhanced desorption negates the need for external heating sources, thereby conserving energy and streamlining the regeneration process.

Noticeably, the synthesis of BAGY is much simpler and affordable compared to that of other solid desiccants such as zeolites, MOFs, and clays. Further, BAGY is compact, self-supporting, and easy to transport and store, making it a promising candidate for large-scale implementation. In addition, many recently developed sorbents may involve some organic or toxic compounds that contribute to environmental issues. However, sodium alginate employed in this work is harmless and is often used in the food industries as a thickener. Alginate-based sorbents are thus more secure for sustainable use. Conclusively, the developed composite offers a unique, efficient, and sustainable alternative for generating clean water from atmospheric air and holds great promise for addressing the critical issue of water scarcity in communities around the world.

## 4 Conclusions

A tailored atmospheric water harvester with superior efficiency that operates over a variety of RHs is reported. The design of BAGY synergistically combines PPyCl, the hydrophilic network of alginate, and photothermal particles (GO) to promote a fast water release under solar irradiance. BAGY exhibits an outstanding water sorption capacity of 7.1 g_w_/g_s_ at 90% RH and 0.91 g_w_/g_s_ at a low RH of 20% at 23 °C. The sorbed water can be released under one sun irradiance without the need for artificial energy source by increasing the surface temperature of the developed networks up to 71 °C, resulting in sustainable water collection with efficiency of 74%. BAGY displays a constant water uptake and stability upon recyclability testing. Thus, this composite offers a unique, efficient, and sustainable solution for producing clean water of high quality and free of pollutants from variable atmospheric air conditions.

## Supporting Information Available

Comprehensive analysis and discussions of FTIR, XRD, XPS, EDS, TGA, and DSC characterizations; sorbent optimization; sorption modeling; optimization of bed configuration; and energy balance in the beads (PDF)
