# Supplementary material for: A Semi-Interpenetrating Network Sorbent of Superior Efficiency for Atmospheric Water Harvesting and Solar-Regenerated Release
Source: ACS Appl Mater Interfaces. 2024 May 8;16(20):26142–52. doi: 10.1021/acsami.4c02451 (PMC11129109; doi:10.1021/acsami.4c02451)
Supplement: Supplementary file 1 — am4c02451_si_001.pdf [file am4c02451_si_001.pdf]

Supporting Information

A semi-interpenetrating network sorbent of superior efficiency for atmospheric water harvesting and solar-regenerated release

Samar N. Abd Elwadood[1,2], *Andreia S. F. Farinha*[3], *Yasser Al Wahedi*[4], *Ali Al Alili*[5], *Geert-Jan Witkamp*[3], *Ludovic F. Dumée*[1,6,7*], *and Georgios N. Karanikolos*[8,9*]

[1]Department of Chemical Engineering, Khalifa University, Abu Dhabi, UAE

[2]Center for Catalysis and Separations (CeCaS), Khalifa University, Abu Dhabi, UAE

[3]King Abdullah University of Science and Technology (KAUST), Water Desalination and Reuse Center (WDRC), Division of Biological and Environmental Science and Engineering (BESE), Saudi Arabia

[4]Abu Dhabi Maritime Academy, Abu Dhabi Ports, Abu Dhabi, UAE

[5]DEWA R&D Center, Dubai Electricity and Water Authority (DEWA), Dubai, UAE

[6]Center for Membranes and Advanced Water Technology (CMAT), Khalifa University, Abu Dhabi, UAE

[7]Research and Innovation Center on 2D nanomaterials, Khalifa University, Arzanah Precinct, Sas Al Nakhl, Abu Dhabi, UAE

[8]Department of Chemical Engineering, University of Patras, Patras, Greece

[9]Institute of Chemical Engineering Sciences, Foundation for Research and Technology-Hellas (FORTH/ICE-HT), Patras, Greece

*Corresponding authors Emails: ludovic.dumee@ku.ac.ae; karanikolos@chemeng.upatras.gr

S1 Supplementary Characterization
S1.1 Morphology (SEM and EDS)
S1.2 Surface area analysis and porosity characteristics
S1.3 Structural analysis (XRD)
S1.4 Chemical interactions (FTIR)
S1.5 Chemical interactions (XPS)
S1.6 Mechanical and rheological properties
S1.7 Thermal stability (TGA)
S1.8 DSC analysis
S1.9 Raman spectroscopy
S1.10 Light/heating absorption kinetics
S1.11 UV-VIS- NIR spectroscopy
S1.12 Moisture capture optimization
S1.13 Sorption mechanism and isotherm modelling
S1.14 Estimation of isosteric heat of sorption
S1.15 Energy transfer/balance during water capturing
S1.16 Energy balance in the beads during desorption under sun radiation
S1.17 Sorption kinetics
S1.18 Optimization of bed configuration and cycle time
S1.19 Water collection experiments
S2 Supplementary References

S1 Supplementary Characterization

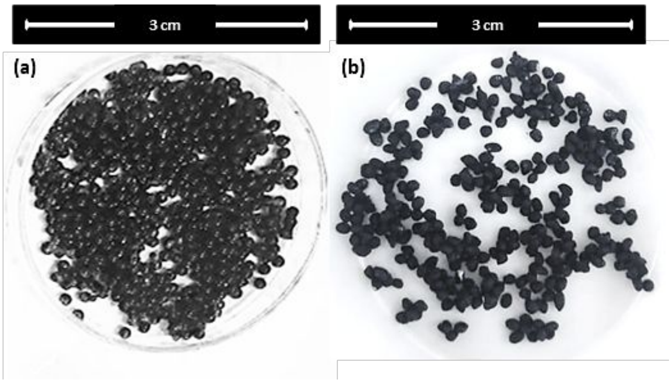

Figure S1 BAGY beads (a) before drying displaying their uniformity as-synthesized and (b) post-drying, revealing their maintained structural integrity and texture.

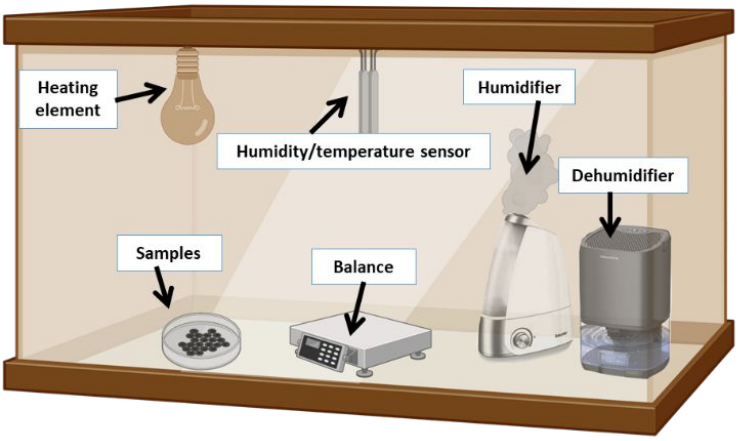

Figure S2 Water sorption test set up (humidity box)

S1.1 Morphology (SEM and EDS)

The morphology of the composites was analyzed using SEM images before and after the addition of PPyCl to the alginate matrix. The SEM imaging of GO (Figure S3 (a)) exhibits crushed sheets and a lamellar surface. The existence of $CaCl_2$ and LiCl in the composite was demonstrated by energy dispersive X-Ray spectroscopy (EDS) and X-Ray diffraction (XRD). Elements such as carbon (C), oxygen (O), chloride (Cl), nitrogen (N), and calcium (Ca) can be seen in Figure S3 (b)owing to the existence of PPyCl, GO, $CaCl_2$, and LiCl in BAGY.

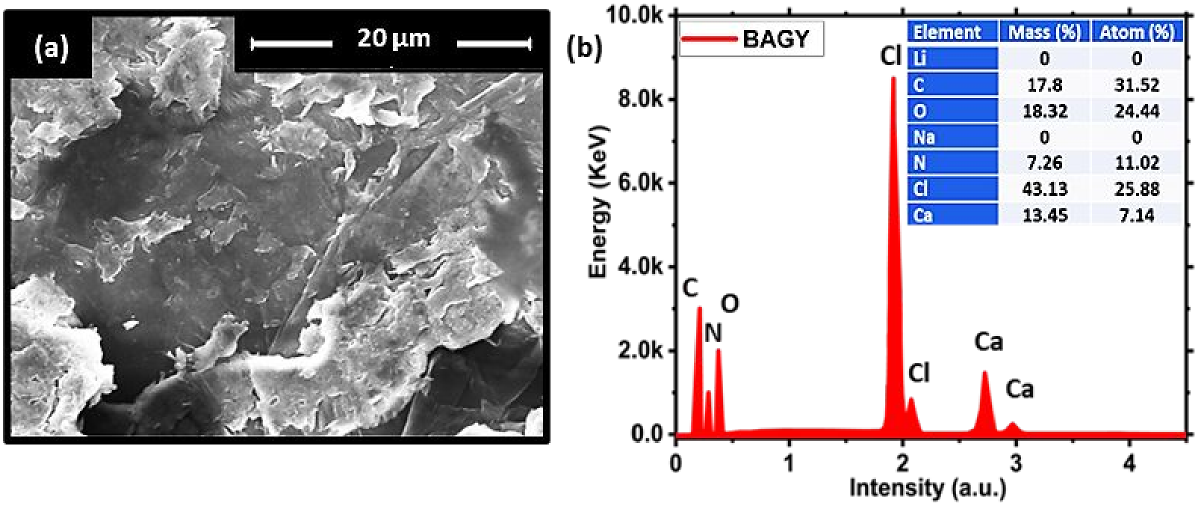

Figure S3 (a) SEM images of GO and (b) EDS spectra and atomic analysis of BAGY.

S1.2 Surface area analysis and porosity characteristics

The textural properties of PPyCl, BAG, and BAGY, including pore size, surface area, and pore volume, were measured using a Micromeritics 3Flex analyzer. PPyCl has a low BET surface area of 3.7 m^2/g, as shown in Table S1, however, BAGY revealed a type II isotherm and exhibited larger surface area (37.7 m^2/g). The specific surface area of BAG was 12.2 m^2/g, which is a larger value than pure crosslinked binary alginate (~4 m^2/g), which can be due to the fact that the porosity of gel was further increased by the addition of GO, while BAGY, which combines PPyCl with alginate and GO, had a higher surface area than BAG The latter is beneficial as typically, the greater the specific surface area, the higher the adsorption capacity.[1] However, the pore size and volume are also important factors in enhancing the adsorption performance.[2] The textural characteristics of the composite sorbents are summarized in Table S1.

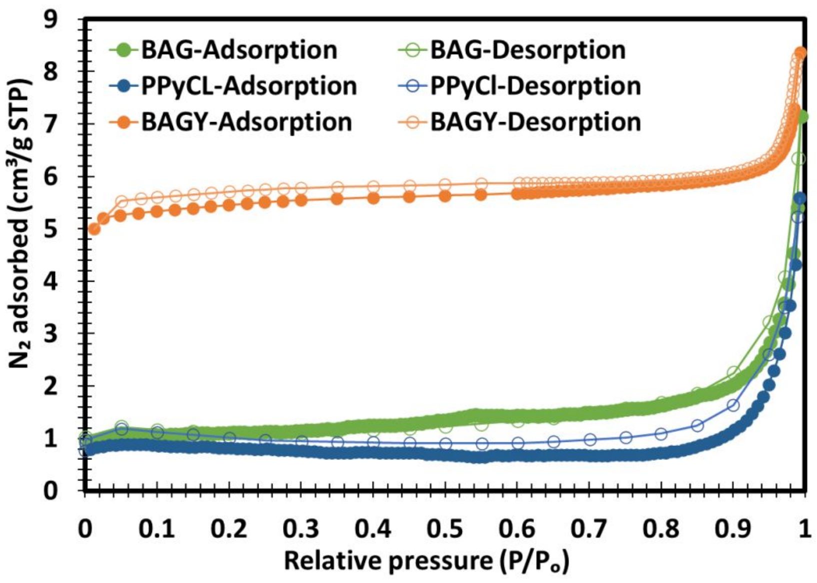

Figure S4 N_2 adsorption-desorption isotherms at 77 K of PPyCl, BAG, and BAGY.

Table S1 The textural parameters of PPyCl and composites obtained by BET analysis.

	PPyCl	BAG	BAGY
**BET Surface Area (m²/g)**	3.7 ± 0.08	12.2 ± 0.7	37.7 ± 0.8
**Pore Volume (cm³/g)**	0.008 ± 0.001	0.07 ± 0.001	0.11 ± 0.002
**Average pore diameter (Å)**	93 ± 1.9	93 ± 2.7	72 ± 1.4

S1.3 Structural analysis (XRD)

Figure S5 (a) displays the XRD patterns for GO, $CaCl_2$, and LiCl. It was possible to see the typical GO peak at 11.6°, which corresponds to the (001) crystal plane.[3,4] The interlayer spacing of GO was found to be 0.76 nm by using the Bragg's equation, which is greater than that of pure graphite (0.34 nm). The effective functionalization and resulting introduction of oxygen functional groups on the graphene sheets during the oxidation process has resulted in the rise in interlayer space.[5,6]

According to the XRD patterns shown in Figure S4, SA and PPyCl are amorphous polymers, which is consistent with previous studies,[7,8] yet, the PPyCl pattern exhibits a wide peak between 25 and 35°, which is attributed to an interplanar d-spacing (3.45 Å) and the π-π interaction of the polypyrrole chain.[9] No new distinctive diffraction peaks were seen in the XRD pattern of the surface of the BAGY beads Figure S5 (b), proving the presence of well-crosslinked amorphous alginate and that there are no salt hydrates on the external surface of the beads. However, the XRD pattern of finely ground BAGY (Figure S5 (b)) show characteristic peaks of the salts, which are attributed to residual salt particles distributed in the internal structure after washing, as well as GO. The $CaCl_2$ and LiCl peaks seen in BAGY demonstrate the effective replacement of Na ions in the cavities of alginate by Ca and Li ions.

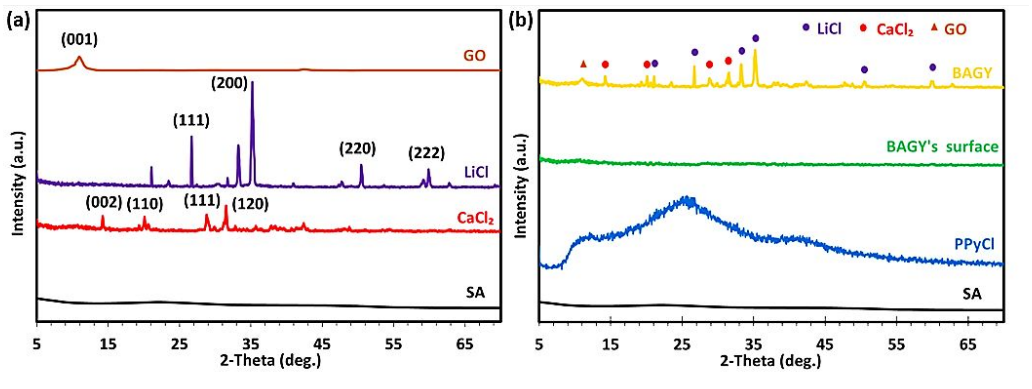

Figure S5 XRD patterns of (a) SA, LiCl, $CaCl_2$ and GO and (b) PPyCl and BAGY.

S1.4 Chemical interactions (FTIR)

Using Fourier transform infrared spectroscopy (FTIR) in the wavenumber range of 4000-400 cm^{-1}, the physicochemical features of PPyCl, salts, alginate, GO, and the resulting composites were examined. PPyCl exhibited three main characteristic bands (Figure S6). The C=C and C-C in-plane bending vibrations of pyrrole rings are witnessed near 1451 and 1533 cm^{-1}.[10] The distinctive band at 780 cm^{-1} demonstrated the PPy ring's C-H deformation vibration.[10] The broad band at 900 cm^{-1} reveals the C-N stretching vibration.[11] Additionally, the C=N stretching and =C-H in-plane vibrations have bands of 1160 cm^{-1} and 1300 cm^{-1}, respectively.[11,12] Also, the band around 1039 cm^{-1} is ascribed to the in-plane deformation of N-H bond.[12] The bands at the expanded region between 3680–2800 cm^{-1} and at 1640 cm^{-1} correlate with the vibration of OH stretching due to the remaining H_2O in the sample, which could be attributed to intra and intermolecular hydrogen-bond interactions of aliphatic and phenolic hydroxyl groups.[10]

From the GO spectra, it is clear that GO is functionalized by a variety of oxygen-enriched groups, including carboxyl, hydroxyl, carbonyl, and epoxy groups.[13] The water molecules that are adsorbed on the surface of GO may be the cause of the wide band across the 3686 to 3000 cm^{-1} range.[14] Characteristic O-C=O groups are found to have distinctive bands at 2083 cm^{-1},[15] while bands at 1720, 1617, 1238, and (1052 and 964) cm^{-1} are linked to stretching vibrations of C=O in carbonyl groups such as esters, ketones, and carboxylic acids on the sheet's edges and defects,[1,15–18] Unoxidized aromatic C=C stretching vibrations in the sp2 carbon skeletal network,[16,17] C-O epoxide stretching,[15] and alcoholic C-O stretching vibrations,[15] respectively.

The spectra of sodium alginate (SA) exhibit a broad band at 3276 cm^{-1}, which is due to O-H stretching vibrations in hydroxyl, carboxylic, phenolic, and chemisorbed water.[7,13,16,17] Additionally, distinctive bands for the -CH symmetric stretching vibrations of the aliphatic -

CH_2 and $-CH_3$ groups are seen around 2902 cm^{-1},[7,16–22] 1610 cm^{-1} for the asymmetric stretching vibrations of –COOH,[7,17,19,20,23] –C-C/C=C,[21] and bending vibration of O-H,[14,24] at 1537 cm^{-1} for tensile vibration of isolated C=C bond [1], at 1415 cm^{-1} for the symmetric -COO stretching vibration,[16,17,19–21,23] at 1390 cm^{-1} for $-CH_2$ (bending),[7] at 1373 -1294 cm^{-1} for C–C bending vibrations,[25] at 1114 cm^{-1} for vibration peak of –C–OH,[22] at 1081-1024 cm^{-1} for C-O-C/ C-O (stretching) vibrations[7,14,20,24,26] with contributions from C–C–H and C–O–H,[18,19,25] at 944 cm^{-1} for the functional groups of guluronic acid,[27] at 808 cm^{-1} or linkage of mannuronic and guluronic units,[25] at 773 cm^{-1} for the functional groups of mannuronic acid,[27] and at 626 cm^{-1} for aromatic rings.[20]

The physicochemical interactions between the alginate, GO, and PPyCl were further examined on the basis of the FTIR results. From FTIR spectra, a strong affinity for water was revealed for BAGY as per the broad hydroxyl band from the polymer backbone between 3250 and 3500 cm^{-1} and that at 1610 cm^{-1} related to the hydroxyl group of structural water (Figure S6 (b)).[28] The intensities of these bands for the BAGY samples were significantly higher compared to SA and PPyCl, confirming the higher water sorption capability.[20] The band corresponding to -OH groups in the crosslinked alginate-based composites shifted to a higher wavenumber as a result of the interactions between the alginate chains forming hydrogen bonds, The formation of these ionic and hydrogen bonds is anticipated to enhance the mechanical properties. After gelation, compared to pure SA, several alginate characteristic bands widened, and their relative intensities altered, demonstrating the presence of potent ionic interactions between the Ca^{2+} or Li^{+} ions and the alginate chains. In general, the substitution of Li^{+} and Ca^{2+} for sodium ions in the alginate structure should not dramatically alter the spectrum of sodium alginate because no new chemical bonds are being introduced into the system. However, the position of carboxylic anion - related bands might reveal the ion exchange process. The attraction between the employed cations and the carboxylic anion varies because

the Na^+, Li^+, and Ca^{2+} have distinct electronegativities.[20] It was possible to see a shifted band with enhanced intensity at 1610 and 1415 cm^{-1} that corresponds to the stretching vibrations of the COO^- groups, symmetrically and asymmetrically. These alterations might be attributed to the production of the carboxylate group during the cross-linking with Ca^{2+} and Li^+, demonstrating a robust crosslinking and interaction between alginate chains and salt cations.[14] In addition to the alginate peaks, a new weak band at 590 cm^{-1} also emerged. This band is ascribed to the nonreacted chloride ions that persisted in the system even after the products had been washed in ethanol. These residual salts also have an impact on the intensify of the peak at 1610 cm^{-1}.[20] The interaction between the components involved during synthesis may be seen in the intensification and modest repositioning of bands below 1500 cm^{-1}, which may be due to the bands of the alginate backbone structure overlapping with the polypyrrole bands.[11,22]

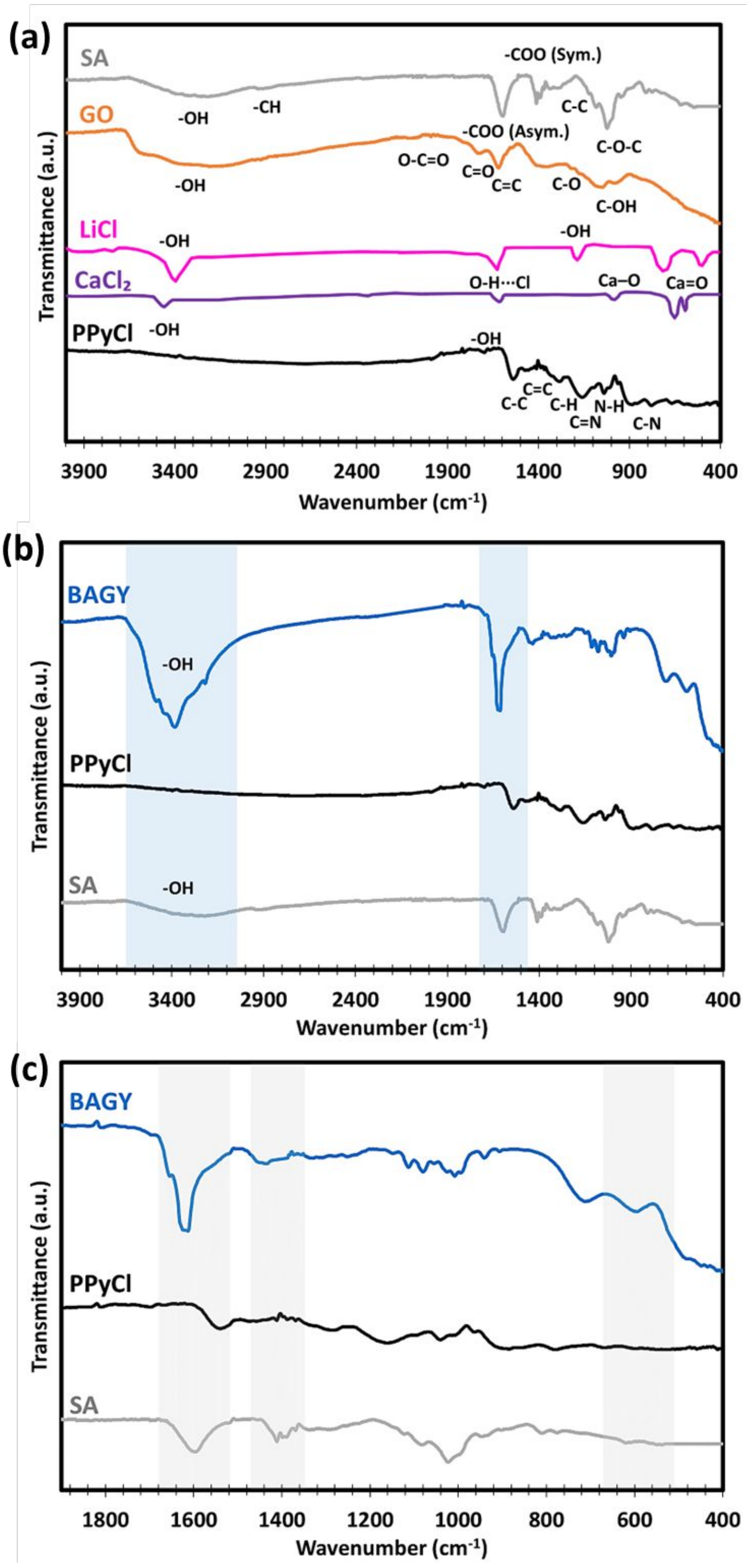

Figure S6 (a) FTIR spectra of PPyCl, SA, GO, LiCl, and $CaCl_2$. (b) Comparison of FTIR spectra of BAGY, PPyCl, and raw SA. (c) FTIR spectra of BAGY, PPyCl, and SA in the 400-1900 cm^{-1} range.

S1.5 Chemical interactions (XPS)

It was determined from the XPS broad scan spectra displayed in Figure S7 that PPyCl and GO were successfully combined inside the BAGY network since its wide scan spectra displayed the key peaks for C 1s, N 1s, Ca 2p, Li 2p, Li 1s, Cl 2s, Cl 2p, and O 1s and their composites with binding energies of 284.8, 400.3, 346.4, 102.4, 55, 266.4, 200.9, and 531.4 eV, respectively.[7,29,30] The peak for C1s can be deconvoluted into four peaks at 284.5, 286.2, 287.3, and 288.6 eV, which correspond to the sp2 domains C-C/C=C/C-H, the sp3 domain C–O/C-O-C bonds, C═O and carboxylic O-C=O, respectively (Figure S8 (b)).[7,29] Additionally, the spectra revealed a prominent O1s peak at 531.4 eV that was caused due to the existence of chemical oxygen species and was linked to three bonds. For instance (in Figure S8 (a)), 531.4 eV for COO^-, 532.6 eV for C=O/C-OH, and 533.2 eV, for hydroxyl bonded to metal (M–OH) (M: Si, Ca) and adsorbed H_2O in the adsorbent, respectively.[31] The N1s peak from the XPS spectra (Figure S8 (d)) was deconvoluted into three components, including -N= at 398.9 eV, -NH- at 400.1 eV, and $-NH^+-$ at 402.1 eV. Also, Cl 2p spectrum is presented in Figure S8 (c), showing a peak at 200.9 eV, corresponding to $Cl^-(-NH^+-)$, Cl^- (H_3O^+) and covalent chlorine species (-Cl) [30]. The PPyCl produced may then be hypothesized to be oxidized and the Cl to be bonded to the PPy chain into a doped-state retained within the PPyCl-alginate composite.[30] Moreover, the peaks at 347.4 and 350.9 eV in Figure S8 (c) were attributed to Ca 2p3/2 and Ca2p1/2, respectively, showing that the calcium atom was bivalent. The findings supported the existence of alginate, GO, salts, polypyrrole, carboxyl, epoxy, Cl, pyrrole N, and Ca components in the composite beads.

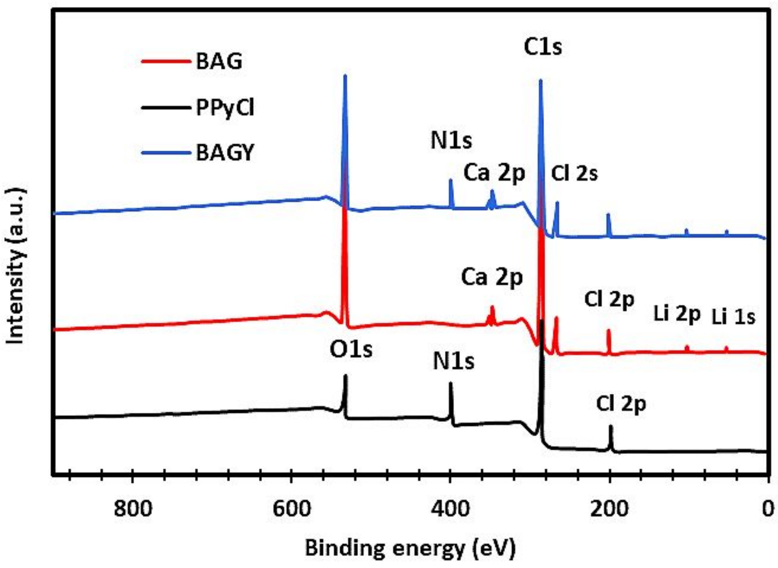

Figure S7 XPS wide spectra of PPyCl, BAG, and BAGY.

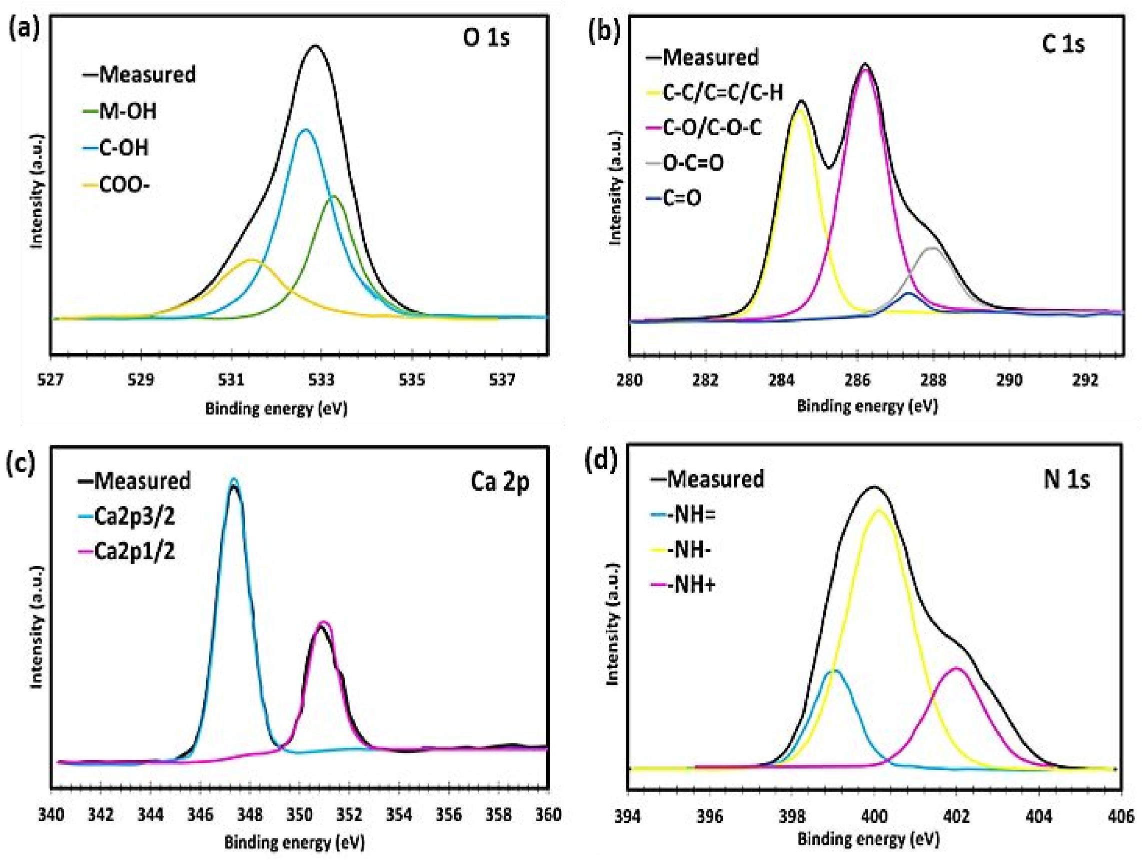

Figure S8 XPS spectra of (a) O 1s, (b) C 1s, (c) Ca 2p, and (d) N 1s.

S1.6 Mechanical and rheological properties

Viscoelasticity is an essential feature of hydrogels and is often tested using an oscillation mode rheometer. The linear viscoelastic zone must first be identified by amplitude scanning since the characteristic constants of the hydrogel can only be acquired in this region during rheological investigation. Because polymeric hydrogels are viscoelastic materials, the addition of an additive might change their mechanical characteristics.[30,32] The storage modulus (G′) and loss modulus (G″) of the gel network, respectively, show how much energy is held therein and how much is lost due to oscillatory stress. All gels exhibit a large linear viscoelastic zone in the dynamic frequency sweep studies. BAG shows identical G′ and G″ values with pure crosslinked alginate (BA), which is attributed to the similar skeleton structure brought by the continuous and flexible polymeric network of the alginate ((Figure 2 (c)). Meanwhile, the G′ values of BAGY are lower than those of BAG and BA, indicating a weaker skeleton, which suggests fewer crosslinking points in comparison to the BA and BAG samples.

Since the wet hydrogels held a lot of water, their compressive stresses were low compared to the dry samples (0.33, 0.59, and 0.51 MPa at compressive strains of 40, 35, and 35 % for BA, BAG, and BAGY, respectively), as shown in Figure S9. In contrast, the dried beads showed high compressive strengths of 3.55, 7.21, and 6.46 MPa for BA, BAG, and BAGY, respectively, with compressive strains of 40, 32, and 31%. Furthermore, the compressive modulus values of dry samples (0.23, 0.86, and 0.45 MPa for BA, BAG, and BAGY, respectively) agreed with the values of compressive stresses.

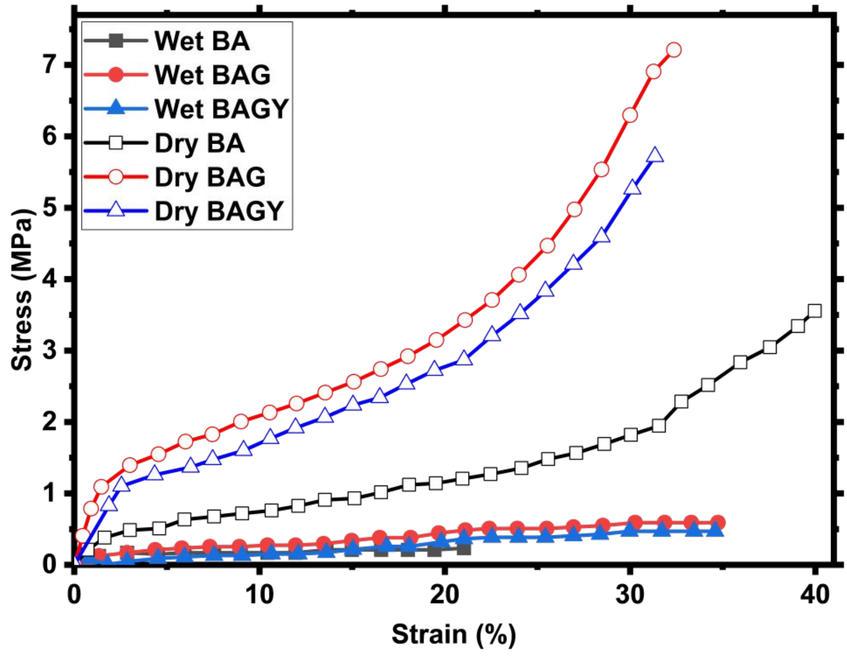

Figure S9 Stress-strain curves of BA, BAG and BAGY.

S1.7 Thermal stability (TGA)

Figure S10 (a) shows the TGA profiles of PPyCl, SA, $CaCl_2$, and LiCl, and how salts undergo dehydration in three steps from dihydrate, monohydrate, and then anhydrous salt. This process is finished at around 190 °C. The graph shows that PPyCl loses considerable weight (9-10 wt.%), which confirms its high hydrophilicity responsible for moisture absorption from the air. Previous research has indicated that PPyCl absorbs around 10 wt.% of water from air.[33] Additionally, the TGA graph in Figure S10 (a) illustrates that the weight loss of sodium alginate (SA) reaches 18% in the temperature range of 50 °C to 250 °C. This weight loss is mainly due to the dehydration of the material and the volatilization of other volatile substances. Only a major considerable pyrolysis stage was found to happen at 250-300 °C, which is caused by the degradation of the main molecular chain of alginate.

The structure of alginate-based polymeric composites contains double bonds, which makes them more vulnerable to high temperatures.[20] The thermal behavior of BAG and BAGY shows a two-step process (Figure S10 (a)). The first stage is the evaporation of water on the surface and trapped inside the inner structure, which may be up to 120–130 °C. Compared to a BAG sample, the TGA profile of BAGY showed a slight increase in the residual weight after the test, indicating that the thermal stability of the BAGY sample has improved after including PPyCl in the network (Figure S10 (b)).

Drying the BAGY samples up to 120 °C did not damage the polymer structure as confirmed by TGA analysis. These samples can be dehydrated up to 120-130 ℃ in a short heating time, but when the desorption time is prolonged, they can be completely dehydrated at 60-80 ℃. A minor weight loss of 1-3% can be observed due to extra dehydration under the testing conditions, which may be extended to 180-190 °C because of the presence of residual salt hydrates within the polymeric matrix. The second stage of weight loss, 10-15% between

190°C to 230°C, is due to the disintegration of glycosidic bonds, the decomposition of alginate, and the depolymerization resulting in the breaking of C-O and C-C bonds.[25,26] At temperatures up to 400°C, pyrolysis of oxygen-containing groups in both GO and alginate occurs,[34] mostly the carboxylic groups from the carbon skeleton, occurs, which leads to the release of CO_2, H_2O, and other small molecules and contributes to mass loss.[13]

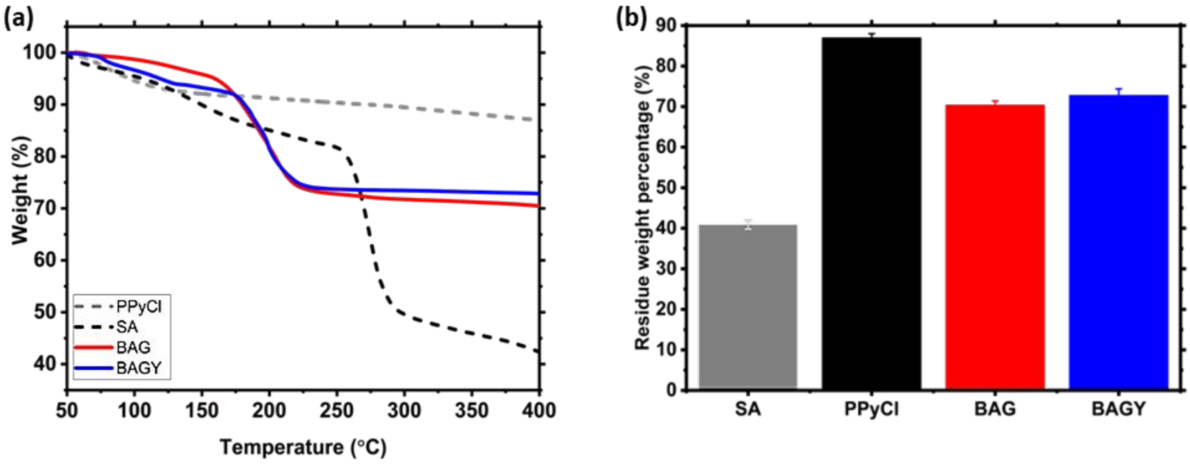

Figure S10 (a) TGA profiles of PPyCl, SA, salts, and the developed composites, and (b) residual weight of composites at the terminal temperature of the TGA analysis.

S1.8 DSC analysis

The DSC heating and cooling curves of all samples reveal the occurrence of a peak for melting that is endothermic and a peak for solidification that is exothermic (Figure S11). BAGY has melting and solidification temperatures higher than 480 °C. Additionally, for alginate-based samples, endothermic bands between 200°C and 250°C and at 450°C are linked to the degradation temperature of the alginate and GO backbone,[16] This confirms the results from TGA that all samples have high stability below 200°C and that PPyCl plays a role in enhancing the thermal stability.[25]

The DSC heating curves show irreversible peaks, which vanish in the cooling curves, indicating that they are likely caused by moisture desorption and irreversible degradation. As opposed to that, the reversible peaks correspond to phase transition states or phase changes. So, DSC analysis can be used to qualitatively evaluate the evaporation enthalpy for all samples and to demonstrate how temperature affects the adsorbed water vapor. As seen in Figure S11, the vaporization enthalpy of PPyCl is 3489 J/g at ~126 °C, and that of BAGY is 3945 J/g at ~88 °C.[10]

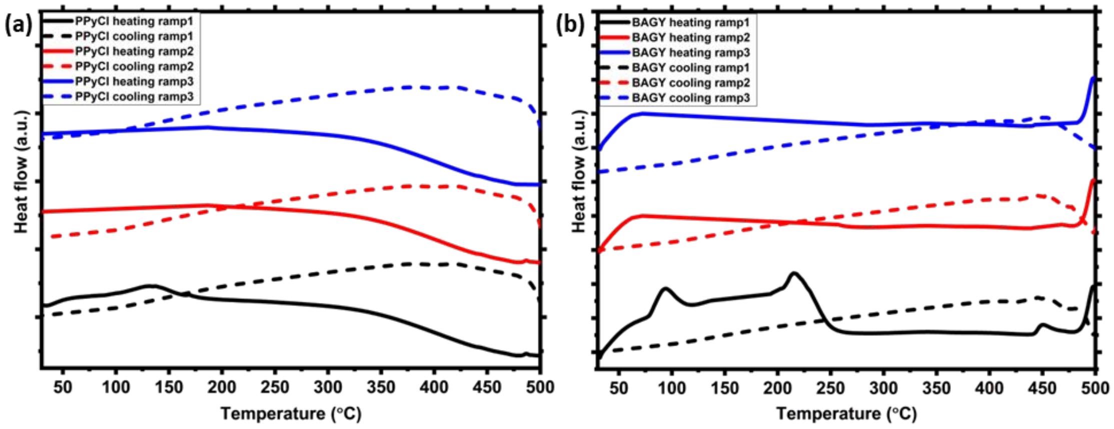

Figure S11 DSC heating and cooling curves of (a) PPyCl and (b) BAGY.

S1.9 Raman spectroscopy

According to Figure S12, the disorder-induced D band at 1330 cm^{-1} and G band at 1602 cm^{-1} can be observed. The presence of the D band is caused by the disorder of the sp2 carbon network, while the presence of the G band is related to the graphitic hexagon-pinch mode, which confirms the presence of GO in the sample.[14] The Raman spectrum of polypyrrole is affected by the excitation wavelength due to the resonance or near-resonance conditions that come from the localized structures formed within the polymer. These effects, which may be linked to the increasing double-bond character of the Cα-α, and Cβ-β bonds, and the decreasing electron density along Cα-β, can be seen in the shifts and the relative intensities of the absorption bands and their positions. Figure 2 (a) displays the Raman spectrum of polypyrrole. The peaks at 1576, 1471, 1314, 1063, and 980 cm^{-1} confirm the presence of polypyrrole, with the peaks representing C=C, C-C, C-N, C-H, and the aromatic ring, respectively. This indicates the composition of the polypyrrole, and verifies the synthesized material.[35] Additionally, the peaks at 980 and 1063 cm^{-1} with the quinonoid polaronic structure indicate the presence of the doped PPy structure.[36]

The BAG sample showed peaks with a shift in the D band to 1335 cm^{-1} and G band to 1605 cm^{-1} after collecting water from the air, further confirming the successful addition of GO into the alginate matrix (Figure S12). The intensity ratio of BAG (ID/IG = 1.08) is almost the same as that of GO (ID/IG = 1.02), indicating that the number of defects and oxygen functionalities in GO stayed unchanged.[14] This can be explained by the fact that incorporating GO into the alginate matrix did not cause any noticeable destruction to the sp2 carbon network of GO.[37–39]

The Raman spectrum of the BAGY (Figure 2 (a)) also shows characteristic vibrational peaks of PPyCl. However, it is clear that the spectra of the PPyCl and BAGY samples are

similar, and no obvious bands of GO are observed in the composite's spectrum. This suggests that the strong interaction between PPyCl and GO may result in the disappearance of GO's characteristic bands.[36] The Raman spectra feature a broad peak in the range of 3050-3630 cm^{-1} that corresponds to vibrations of hydrogen bonds in water. These peaks can be divided into two categories: (1) water molecules that have four hydrogen bonds, which can be seen between 3270 and 3410 cm^{-1}, and (2) water molecules that have weak or broken hydrogen bonds caused by the polymeric network in the composites, which can be seen between 3520 and 3630 cm^{-1}. These peaks can help identify the state of hydrogen bonding in water molecules within a sample.[30] The peak at 3270 cm^{-1} is associated with in-phase vibrations of water molecules in hydrogen-bonded molecules, while the peak at 3410 cm^{-1} is associated with out-of-phase vibrations between the neighboring water molecules and methyl groups. The peaks at 3520 and 3630 cm^{-1} correspond to the stretching of weakly hydrogen-bonded H_2O molecules.[30]

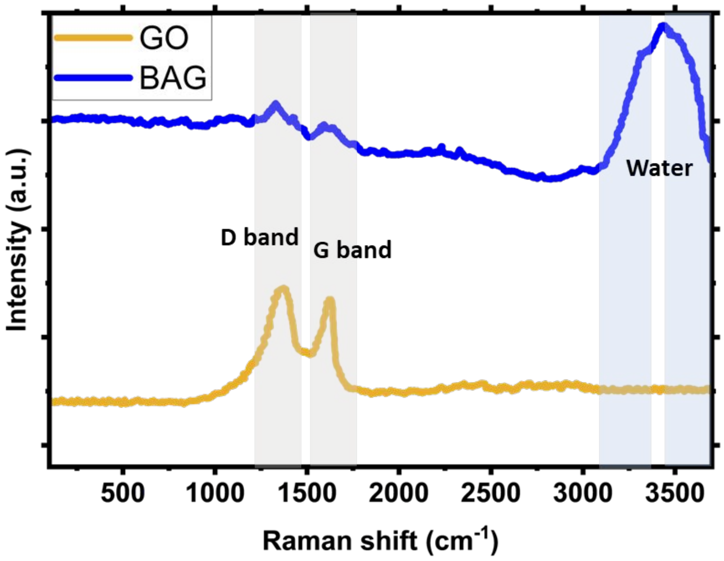

Figure S12 Raman spectra of GO and BAG.

S1.10 Light/heating absorption kinetics

A simulated sunlight source was utilized to assess the samples' ability to convert light to heat since solar radiation provides the thermal energy needed for the desorption process. The surface temperature of BAG, BAY, BAGY, and PPyCl increased by about 30 to 40 °C over the first 10 min, but only by about 9 °C for BA (Figure S12). The temperatures then reached a plateau at 60 °C for BAG, 62 °C for BAY, 58 °C for PPyCl, and 71 °C for BAGY, indicating that the lost energy from surface was in equilibrium with the absorbed solar energy. But after 120 min, the temperature of the BA sample gradually rose to 50 °C and stayed there while being exposed to the same amount of light. The homogenous temperature distribution on the surface of the beads shows that the distribution of PPyCl and GO in the samples is uniform, as confirmed by the IR image in the inset of Figure S13.

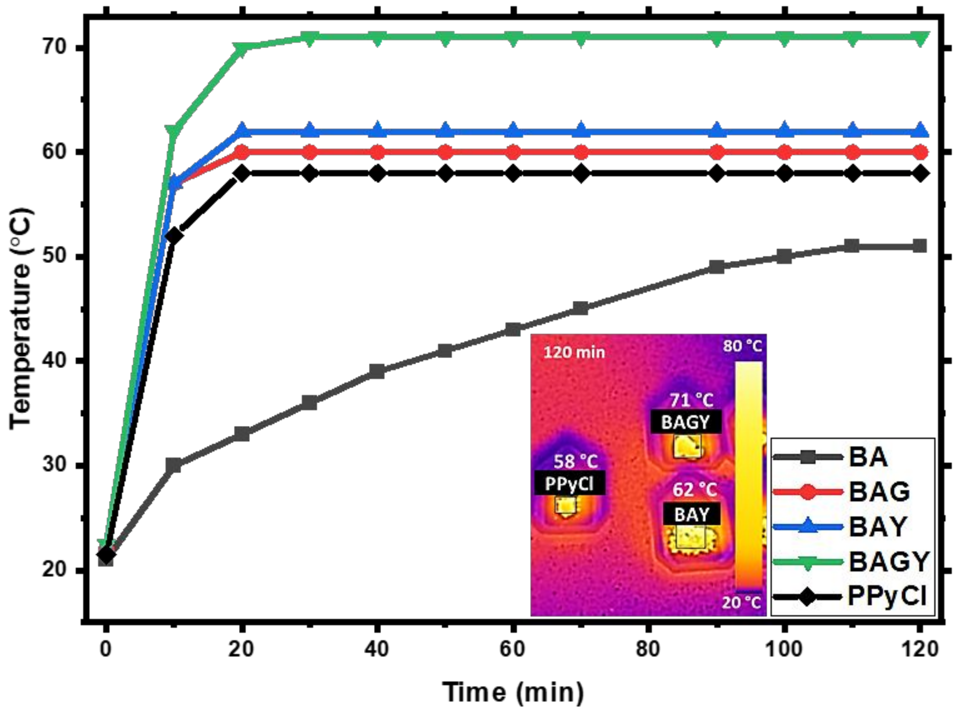

Figure S13 Light and heat conversion kinetics under 1 sun irradiation.

S1.11 UV-VIS- NIR spectroscopy

The efficiency of solar-driven water desorption using a composite material is related to its ability to absorb light and convert it into heat.[20] The composite developed herein contained GO as one of its constituents to enhance this action. When light is absorbed by the composite, electrons are excited to a higher energy state, and when they return to the ground state, nonradiative transitions occur, which produce heat. Research has shown that the near-infrared (NIR) absorbance spectrum of water has strong absorption intensities between 1400 nm and 2500 nm.[29] The narrow absorption wavelength of water makes it challenging to achieve high efficiency in solar-driven water desorption. To improve efficiency, the water sorbent should have increased absorption in the NIR region below 1400 nm, which can compensate for the limited absorption range of water, which corresponds to the broad range of the standard solar spectrum. The ultraviolet-visible-near-infrared (UV-vis-NIR) absorption spectra of the samples were collected over the full spectrum range of 200-1400 nm, which is crucial for effective light harvesting of solar radiation.

Polypyrrole (PPy) is a low-cost, easily synthesized, and biocompatible black polymer that has been widely used in solar evaporation due to its excellent light absorption properties. [10] Graphene oxide (GO) is also incorporated as a photothermal material in the composite due to its strong absorption in the visible light range, which dominates sunlight. The black color of GO also enhances the photo-absorption of the composite samples. The spectral properties of the composites containing PPyCl and GO (5 wt%) and those without them were measured over a wavelength range from 200 to 1400 nm. The results show that the light absorption of BA (alginate without GO) was 54%. However, the PPyCl and PPyCl/alginate (BAY) samples absorbed ~98% and 94% of light respectively, with very little transmittance and reflectance (less than 0.1% and 5%, respectively), as shown in Figure S14. When GO was incorporated

with PPyCl in the BAGY sample, the light absorbance increased from 94% to ~98%. Based on the results, the BAGY is expected to have efficient optical absorption across the spectrum. Consequently, the heat created was efficiently contained inside and dissipated through the GO and PPyCl particles with little heat loss to the air, which improved the evaporation of water.

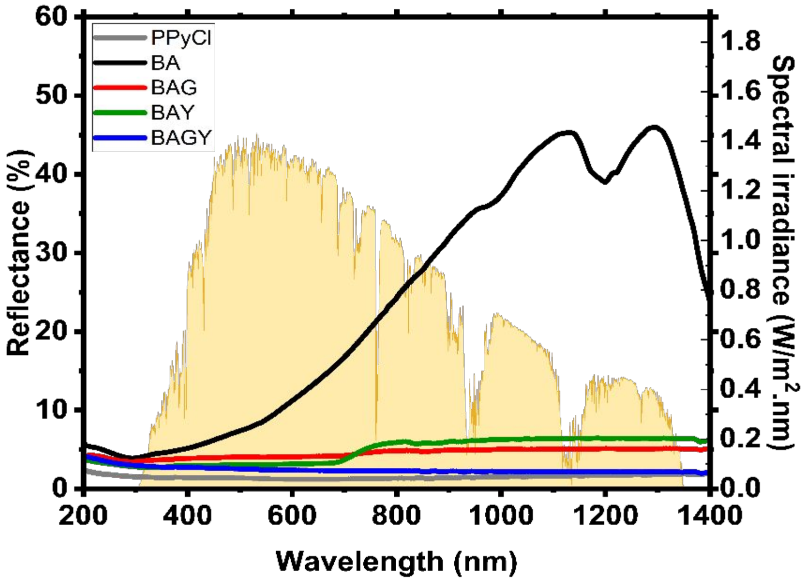

Figure S14 UV-vis-NIR reflectance spectra of the PPyCl and alginate crosslinked composites.

S1.12 Moisture capture optimization

A screening strategy was followed to optimize the water uptake of the developed composite. Sodium alginate concentrations were varied across different internal (M/G) ratios, assessing their impact on moisture capture (Figure S15). All details for sample compositions are presented in Table S2. For all M/G ratios and SA concentrations, this optimization procedure identified optimal alginate concentrations for each M/G ratio, notably 1.23 wt.% for an M/G ratio of 1:2, demonstrating the highest water uptake. Additionally, we investigated the role of salt solution saturation in the crosslinking process, finding that higher salt concentrations in the gelation solutions enhanced water sorption by retaining more residual salt within the beads (Figure S16). We also examined the effects of varying polypyrrole chloride (PPyCl) to sodium alginate (SA) ratios on water sorption, as shown in Figure S17. This allowed us to pinpoint the sample configurations with the best performance under varying RH conditions.

Table S2 Amount and compositions of samples with different SA concentrations and M/G ratios.

Code name	A1a	A1b	A1c	A1d	A0.5a	A0.5b	A0.5c	A0.5d	A2a	A2b	A2c	A2d
**Water amount (g)**	40	40	40	40	40	40	40	40	40	40	40	40
**M/G ratio**	1:1	1:1	1:1	1:1	1:2	1:2	1:2	1:2	2:1	2:1	2:1	2:1
**SA amount (g)**	0.5	1	2	3	0.2	0.5	0.8	1	0.2	0.5	0.8	1
**Concentration wt.%**	1.23	2.43	4.76	6.97	0.49	1.23	1.96	2.43	0.49	1.23	1.96	2.43

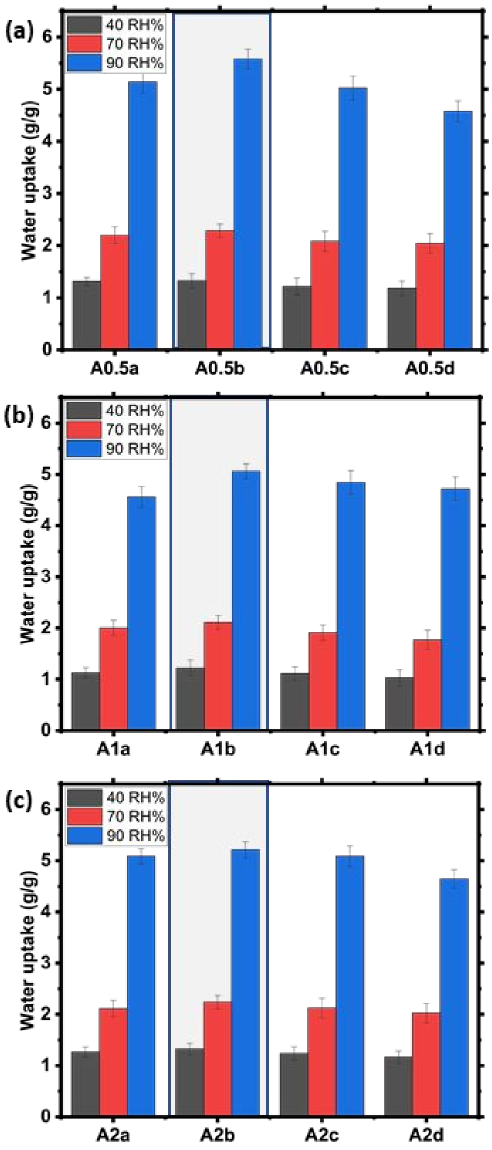

Figure S15 (a) Dependence of water uptake on SA concentration with M/G ratio of 0.5, (b) Dependence of water uptake on SA concentration with M/G ratio of 1, and (c) Dependence of water uptake on SA concentration with M/G ratio of 2.

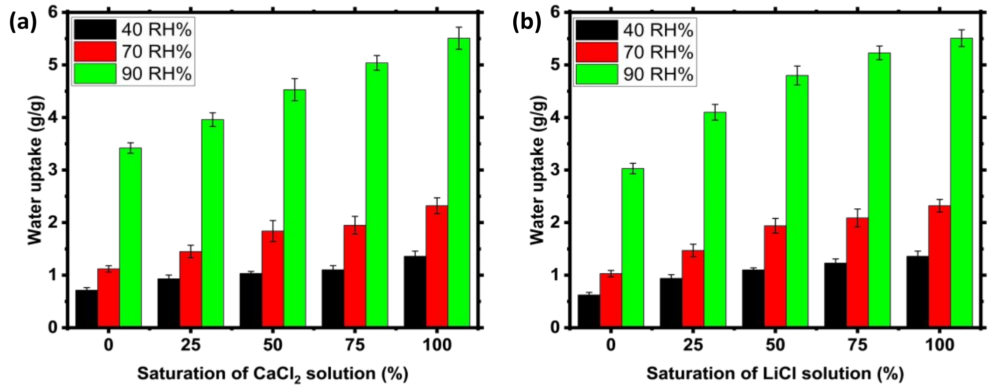

Figure S16 The impact of saturation percentage of gelation salt solutions on BAGS water absorption: (a) $CaCl_2$ solution and (b) LiCl solution.

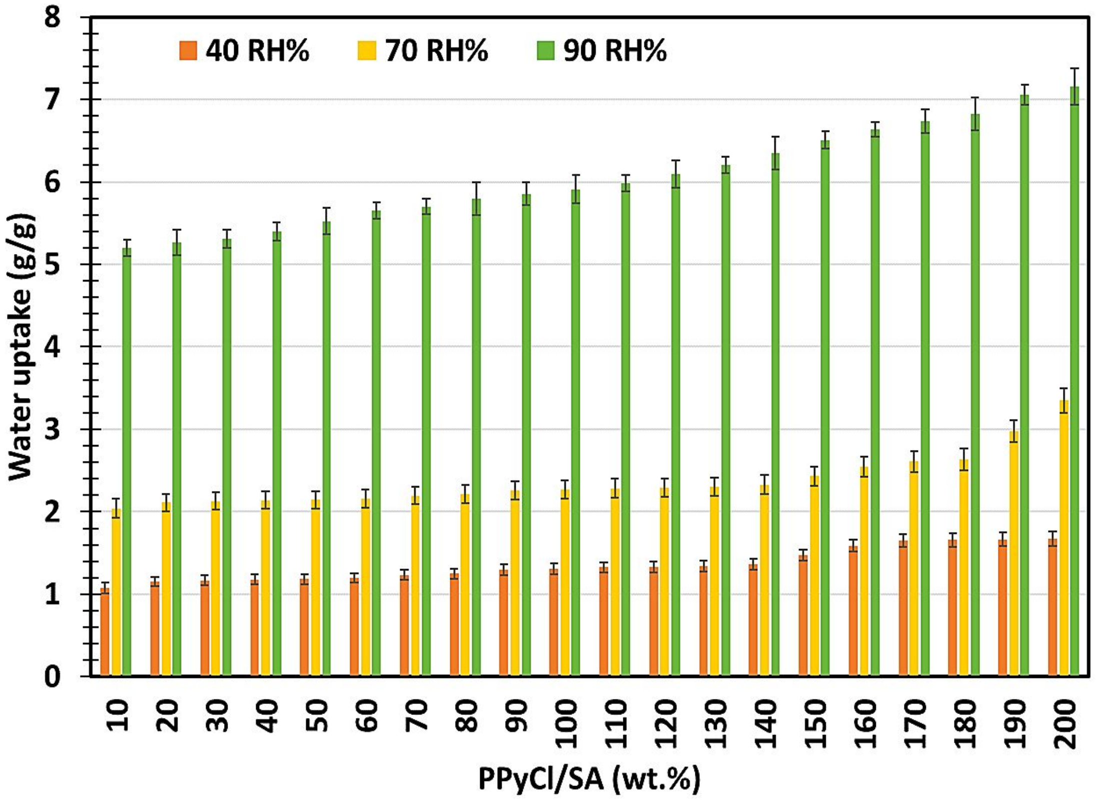

Figure S17 Dependence of water uptake on PPyCl/SA relative concentration ratio at 40, 70, 90% RH at room temperature.

S1.13 Sorption mechanism and isotherm modelling

To fit the experimental results of moisture sorption on BAGY, four adsorption isotherm models were used: Langmuir (Figure S19 (a)), Freundlich (Figure S19 (b)), Frenkel-Halsey-Hill (FHH) (Figure S19 (c)), and Guggenheim-Anderson-de Boer (GAB) (Figure S19 (d)). The parameters of each model are provided in the Table S3. The Langmuir model was found to be inadequate for describing the isotherm of BAGY, while the Freundlich model, which considers adsorption on uneven and diverse surface sites, provided a moderate match. Based on the high Adj.R^2 correlation coefficient values, it was determined that the FHH and GAB isotherms were the most suitable for fitting the experimental data of BAGY. The FHH model is commonly used to describe the sorption of water vapor on clay particles, and it was found that the water vapor sorption pattern for the BAGY is similar to that for clays.[40] The FHH model assumes that when the relative humidity is high, capillary condensation and the presence of multiple layers within the material cause water to fill the internal structure through hydrogen bonding and Van der Waals interactions.[41,42] The FHH model posits that a gradient of sorption potential exists and is determined by the distance between the surface of the sorbent and the sorbate layer.[43] The BAGY's experimental data being consistent with the FHH model, indicates the presence of capillary condensation and enhanced interactions between water molecules and sorption sites at high relative humidities. The model was also used to calculate the single-layer adsorption capacity (q_m), and it was found that these values were higher at lower temperatures and decreased as temperature increased, supporting that water uptake is greater at lower temperatures.

Additionally, the experimental data was found to have a strong correlation with the GAB model. The GAB model can be used to calculate the amount of coverage of the sorbate molecules, known as bounded water, by determining the monolayer adsorption capacity (q_m).

It was observed that at lower temperatures, q_m had a higher value, which decreased as the temperature increased. Additionally, the values of the parameter c_G, which indicates the presence of linkage between the sorbate and adsorbent, decreased with increasing temperature.

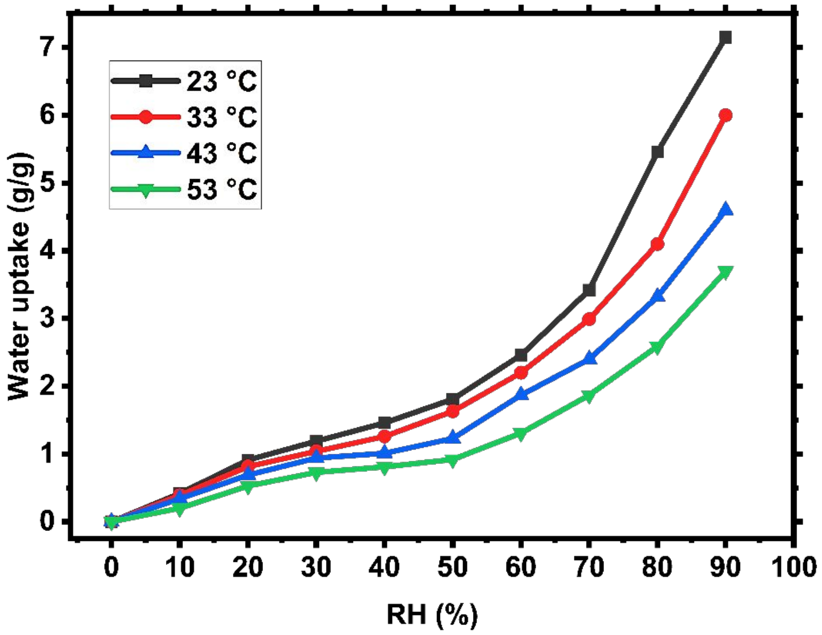

Figure S18 Water vapor sorption isotherms of BAGY at 23, 33, 43, and 53 °C.

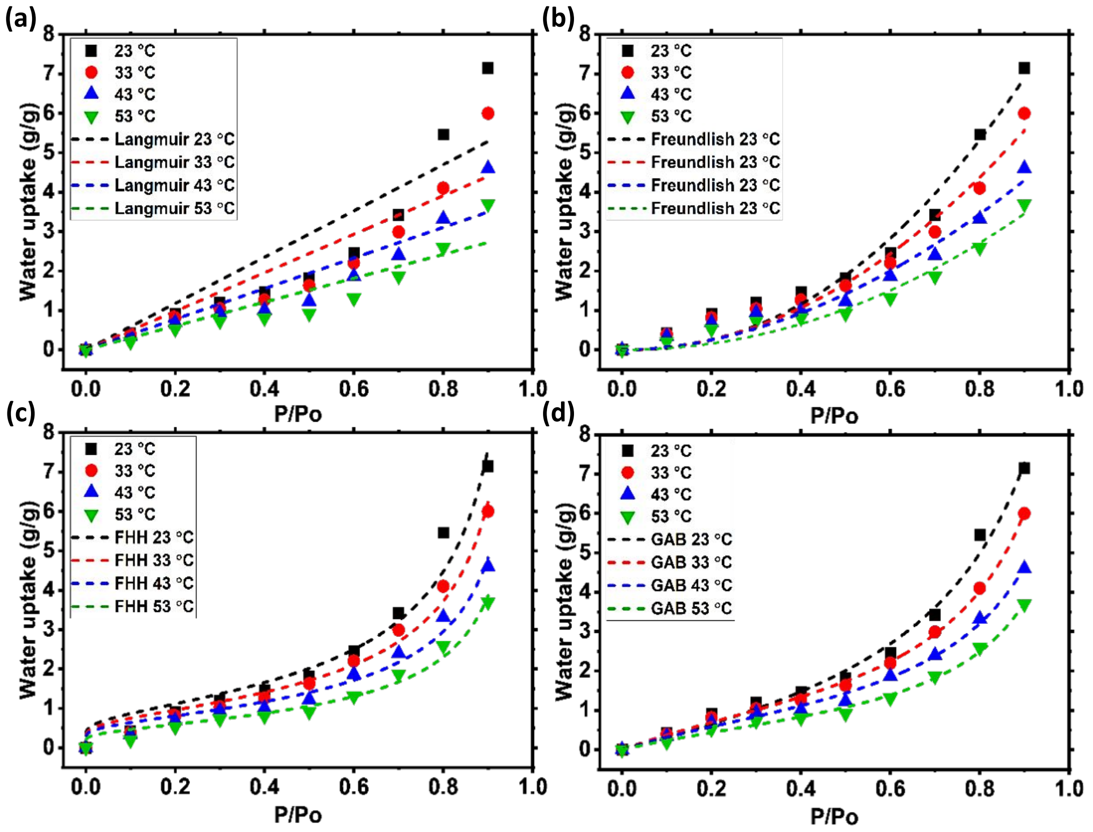

Figure S19 Fitting of experimental isotherm data to (a) Langmuir, (b) Freundlich, (c) FHH, and (d) GAB isotherm models.

Table S3 Different isotherm model parameters for the water absorption on BAGY.

Isotherm model	Parameter	23 °C	33 °C	43 °C	53 °C
**Langmuir**	**q_m (g/g)**	29149	21956	15841	13700
	**b**	2.01×10^{-4}	2.22×10^{-4}	2.45×10^{-4}	2.2×10^{-4}
	**Adj.R^2**	0.80	0.82	0.85	0.82
**Freundlich**	**K_f (L/mg)$^{1/n}$(mg/g)**	8.69	6.89	5.24	4.28
	**n**	0.45	0.49	0.53	0.48
	**Adj.R^2**	0.96	0.96	0.96	0.95
**FHH**	**q_m (g/g)**	1.55	1.32	1.10	0.82
	**r**	1.42	1.45	1.52	1.45
	**Adj.R^2**	0.96	0.98	0.97	0.97

**GAB**	**q_m (g/g)**	2.04	1.30	1.10	0.79
	**k_G**	0.84	0.88	0.86	0.88
	**c_G**	1.78	3.64	3.73	3.80
	**Adj.R^2**	0.98	0.99	0.99	0.99

S1.14 Estimation of isosteric heat of sorption

The isochoric heat of sorption (ΔH_s) was determined using the Clausius-Clapeyron equation (Equation S1):[44]

$$\boldsymbol{ln\,(p) = -\frac{\Delta Hs}{RT} + C} \quad \text{(S1)}$$

The Clausius-Clapeyron equation relates the change in heat of adsorption (ΔH_s) to the change in pressure (p) and temperature (T). In this equation, R and T represent the ideal gas constant and temperature, respectively, and C is an integrating constant. To calculate ΔH_s, the sorption isotherms were converted into isosteres by plotting them as ln(p) versus 1/T (Figure S20) for different water vapor uptakes. The slope ($-\Delta H_s/R$) was then used to determine the ΔH_s. Figure 2 (e) shows the graph of ΔH_s at various equilibrium water uptakes, and Table S4 lists the values of ΔHs as well as the corresponding correlation coefficients. The ΔH_s values were found to be much higher at lower sorption capacities because, at lower relative humidity levels, water molecules were strongly bound to the matrix. However, at higher water uptakes and RH levels, most of the sorbed water was present as weakly bonded liquified water, resulting in relatively low ΔH_s values. As the humidity levels increased, clusters of water molecules grew and began to penetrate deeper into the inner structure of the sorbent and fill the pores. This is supported by the fact that the ΔHs values of BAGY were found to be in the range of 65-44 kJ/mol, with values of < 50 kJ/mol for coverage higher than 1 g/g, revealing physical interactions (Table S4 and Figure 2 (e)).

Additionally, the desorption enthalpy was determined by conducting sorption tests using a DSC instrument in combination with a modular humidity generator under a nitrogen purging environment. One of the main assumptions was that the kinetics are same during TGA and DSC experiments. This method allowed for the determination of the enthalpy of desorption

of water vapor for BAGY at different relative humidity levels (10-90% RH). The DSC tests were performed with two temperature ramps to remove the heat history of the material. Firstly, samples were heated to 150 °C and 0% RH at a rate of 1 °C/min, then cooled down to 23 °C with a cooling rate of 10 °C/min. At the end of the first ramp, the sample is considered to be in a dry state with no leftover water present. The enthalpy of sorption was calculated by applying the following equation:[45]

$$\boldsymbol{\Delta H_s = -\frac{Q_{HFS}}{m_s}} \quad \text{(Equation S2)}$$

During these experiments, the thermal energy (Q_{HFS}) measured by the heat flux sensor is related to the amount of vapor sorbed by the sample (m_s). This amount is determined by comparing simultaneous TGA results with the water uptakes obtained from the gravimetric analysis done using the Q5000SA sorption instrument. The data for the enthalpies of sorption and release for BAGY are shown in Table S5.

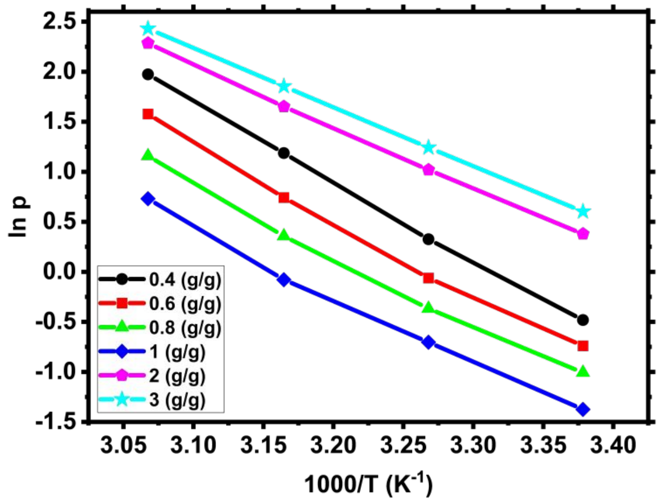

Figure S20 Plots of the ln (p) versus 1/T at various water uptakes.

Table S4 Isosteric heat of sorption parameters based on the Clausius-Clapeyron equation.

Water uptake (g/g)	$\Delta H_s/R$ (K)	ΔH_s (KJ/mol)	ΔH_s (J/g)	R^2
**0.4**	-7.9	65.9	3661	0.99
**0.6**	-7.4	62.1	3450	0.99
**0.8**	-6.9	57.8	3211	0.99
**1**	-6.0	50.3	2794	0.99
**2**	-5.5	46.2	2566	0.99
**3**	-5.3	44.5	2472	0.99

Table S5 Direct measurements of ΔH_s using DSC.

RH (%)	Water uptake (g/g)	ΔH_s (KJ/mol)	ΔH_s (J/g)
**10**	0.4	65.6 ± 1.5	3644 ± 83
**20**	0.9	55.4 ± 1.3	3077 ± 72
**30**	1.1	48.8 ± 0.9	2711 ± 50
**40**	1.5	46.5 ± 1.2	2583 ± 66
**50**	1.8	45.7 ± 2.1	2538 ± 116
**60**	2.4	44.6 ± 1.9	2476 ± 105
**70**	3.4	44.3 ± 3.1	2461 ± 172
**80**	5.4	44.5 ± 1.5	2472 ± 83
**90**	7.1	44.6 ± 1.7	2476 ± 94

S1.15 Energy transfer/balance during water capturing

The process of converting vapor into liquid is exothermic, while the polymer dissolution of endothermic. Due to the high molecular weight, the interaction between polymer chains is strong, and water molecules needs to insert into the spaces between the polymer chains, which requires energy. The balance between these two processes, along with any uncontrolled heat loss to the surroundings, determines the overall temperature change of the sample. The temperature change during moisture sorption is used to confirm this claim. At the beginning of the sorption test, an increase in the temperature of sample has been recorded from 24 to 27 °C as a result of the initiation of the endothermic water sorption (Figure S21). While no temperature change could be observed after some time, suggesting that the overall BAGY's heating or cooling is negligible due to the dynamic equilibrium of thermal energy release and dissipation to surroundings. Dissolution appears not to have occurred, as evidenced by the unchanging weight of the BAGY sample even after several sorption/desorption cycles and the absence of any associated bands in DSC curves, suggesting that the generated energy from sorption was not sufficient to activate the dissolution of the polymer. This might be owing to BAGY's strong tendency for water capture and a lack of heat to stimulate polymer disintegration, thus promoting the composite's stability.

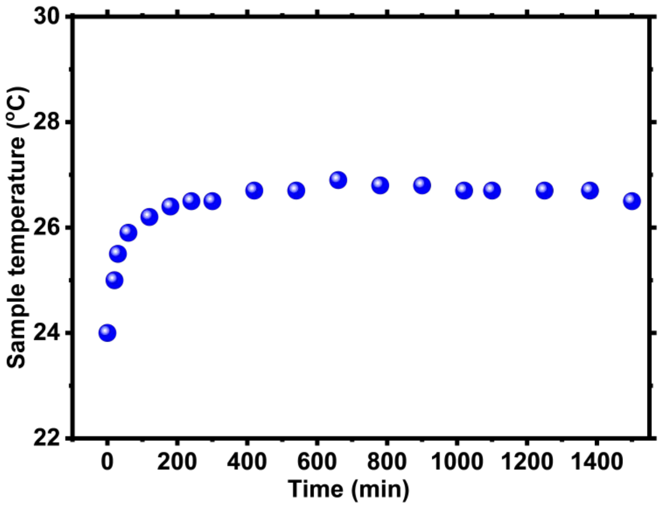

Figure S21 The temperature profile of BAGY during the water capturing process.

S1.16 Energy balance in the beads during desorption under sun radiation

The analysis of heat released from the sorbent when it is exposed to an open environment is presented in Equation S3. The total energy received from the sun equals the energy exchanged through radiation with the environment, energy transferred into the system by convection, heat used for evaporation, and finally, heat conducted (Q_{cond}) to the lower layers.[20]

$$\left(\frac{q}{\alpha}\right)_{sun} \alpha_{sun} = \alpha_{low\ temp}\, \sigma\, (T^4_{surface} - T^4_{\infty}) + h\, (T_{surface} - T_{\infty}) + \dot{m}\, h_{evap} + Q_{cond} \quad \text{(Equation S3)}$$

where α_{sun}, $\alpha_{low\ temp}$, σ, $T_{surface}$, T_{∞}, h, $\dot{m}$ and h_{evap} are absorptivity for solar irradiance, absorptivity of radiation at low-temperature, Stefan-Boltzmann constant, the surface temperature, the temperature of surrounding, the coefficient of convective heat transfer, the mass flux of evaporation, and the enthalpy of vaporization, respectively. The α_{sun} and $\alpha_{low\ temp}$ for black material can be considered equal 97%. $T_{surface}$ and T_{∞} are almost 69 ℃ and 23 ℃, respectively, under 1 sun. Also, h can be considered 5 W/m^2 /K.[20]

$1380 \times 0.97 = 0.97 \times 5.669 \times 10^{-8} (342^4 - 296^4) + 5 \times (342 - 296) + \dot{m}\, h_{evap} + Q_{cond} = 1338.6\ W/m^2$

$\dot{m}\, h_{evap} + Q_{cond} = 778.4\ W/m^2$

The total energy that leaves the system can be separated into three parts: reflection loss (3%), heat loss through convection (17.1%), and heat loss through radiation (21.8%). The remaining energy, which is 58.1%, is used for water evaporation and heat conduction. However, when taking into account the insulation of the bottom face of the box and the energy balance boundaries around the material, heat conduction is not considered a loss from the system. Despite this, the low thermal conductivity causes a high thermal gradient on the surface. This leads to an increased evaporation rate on the surface, resulting in an increase in mass transfer from the core to the surface by diffusion.

S1.17 Sorption kinetics

The system's ability to extract more water from the external environment within a given time due to faster kinetics, which allows for more cycles of moisture capturing to occur in a shorter period of time, was also evaluated. The experiment was conducted by exposing the material to N_2 gas and atmospheric air with varying RH levels (10%, 40%, and 70%) at a temperature of 23 °C in a testing chamber after it had been fully dried. The material was regenerated at temperatures of 60, 70, and 80 °C to study its regeneration behavior over time. The results showed that the material's rate of water uptake was the highest at the onset of the experiment, and then gradually decreased as the sorption process continued (Figure 2 (f) and Figure S22 (a)).

In order to better understand the kinetics of water capture by BAGY, two kinetic models were used, the pseudo-first-order and pseudo-second-order models. The parameters for these models are shown in Table S6 and the results of the experimental dataset fitting are shown in Figure S22 (b). Both models have correlation coefficients greater than 0.9, indicating that they are able to effectively depict the sorption process at different RH levels. Specifically, the pseudo-first-order model showed a correlation coefficient of 0.99 at 40 and 70 RH %, making it an effective model for understanding absorption at these levels. The pseudo-second-order model also had a high correlation coefficient of 0.99 and predicted equilibrium water uptake that was close to the experimental value. This suggests that the pseudo-second-order model accurately captures the behavior of BAGY and that BAGY sorbs water through both physical and chemical sorption.

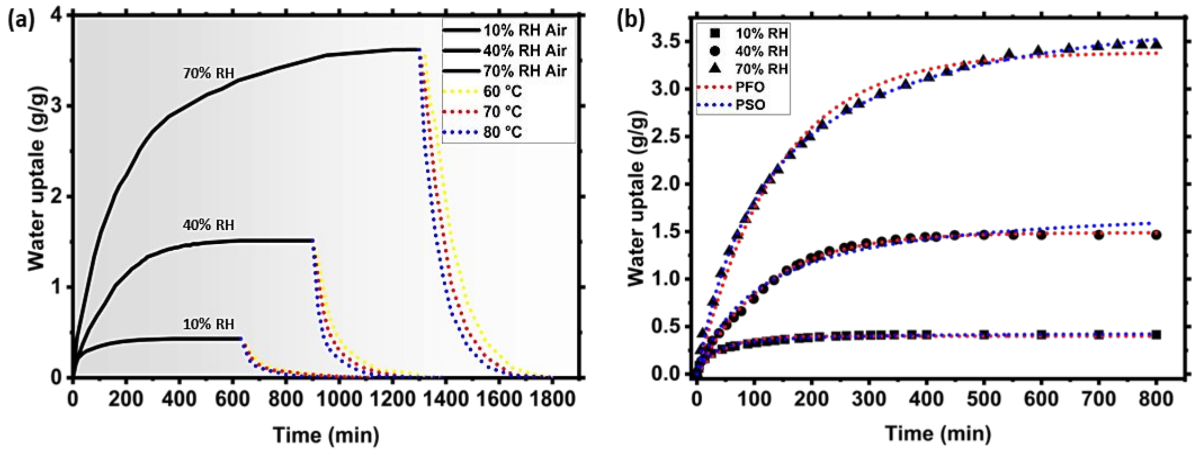

Figure S22 (a) Water uptake/release kinetics measurements for BAGY from atmospheric air at 10, 40, and 70% RH, and (b) plots of fitting of water vapor sorption kinetics on BAGY.

Table S6 Different kinetics parameters of BAGY at 10, 40 and 70% relative humidity.

Model	Parameter	10%	40%	70%
**pseudo-first-order**	**q_e (g/g)**	0.69	1.74	3.01
	**k_1 (min^{-1})**	0.019	0.010	0.010
	**Adj.R^2**	0.94	0.99	0.99
**pseudo-second-order**	**q_e (g/g)**	0.75	2.1	3.4
	**k_2 (g/g.min)**	0.049	0.005	0.003
	**Adj.R^2**	0.97	0.98	0.99

S1.18 Optimization of bed configuration and cycle time

To better understand and design a device for real-world moisture capture application, experiments were conducted to determine the time it takes for BAGY to generate water spills around the sorbent's bead. Two plates of material were used, one with a single layer of BAGY beads and the other with multiple layers. These plates were placed in different RH conditions and were observed for water spill formation to occur. The time of water spill formation and the samples' weight were recorded (Figure S23). At 20% RH, no water spill was observed. The area of each circle in Figure S23 represents the amount of water uptake in g/g of material when water leakage starts. The results show that the single layer structure can collect more vapor, and water spill formation occurs much faster. This can be attributed to the fact that in multiple layers, the bottom layers are not directly exposed to air and the water sorbed by the upper layer passes in the lower layers through the beads via diffusion, thus the latter cannot contribute directly in AWH. These experiments suggest that BAGY is better used in a single layer configuration. Additionally, cycles can be designed with short humidification times (75, 69, 50, 37 and 20 min for 40%, 50%, 60%, 70% and 80%, respectively) followed by desorption, if water spills are needed to be avoided in real system design to reduce the possibility of subsequent deliquescence. Another approach is to use a porous matrix or conventional desiccant to support the developed composite, and any possible spilled water can be sustained in the sorbent.

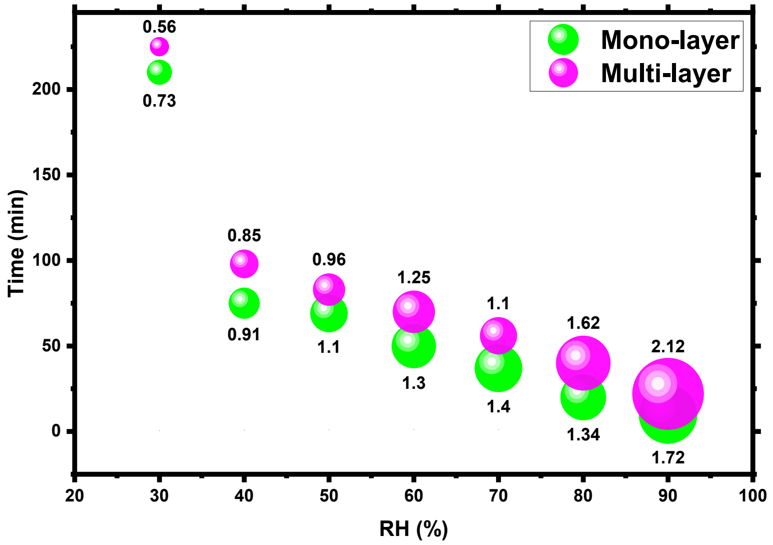

Figure S23 Water spill formation time (y-axis) at different relative humidities and water uptake in g_w/g_{BAGY} (presented by the circles' area) recorded at leakage time in different configurations of bed.

S1.19 Water collection experiments

S1.19.1 Indoor test

A simple prototype was used for harvesting water using BAGY through a passive sorption process. Over time, the system mass and the composite surface temperature experienced changes. Additionally, the water generated by the process was collected. In order to carry out this experiment, sheets with a high reflectivity and low thermal conductivity were used to insulate the sides of the container. This helped to keep the temperature of the side walls lower than the surrounding by shielding them from sun radiation. The device bottom was additionally insulated to prevent heat loss. During the sorption from the ambient air, the simulator shutter remained closed, and the upper lid of the box opened. When the material was exposed to humid ambient air, it collected enough water vapor to become saturated (Figure 3 (a)). When the sorption phase was complete, the amount of sorbed water was measured (2.74 g_w/g_s) and liquid water was seen surrounding the composite beads. The box was sealed to start the desorption and condensation processes under the circumstances of simulated sunlight. Meanwhile, water droplets began to progressively cover the device's wall. The steps involved in creating collectable water may be defined as follows: droplet production, growth, multiplication, condensation, and gravity-assisted accumulation of large clusters at the bottom of the plastic box. Upon completion of the desorption process, the harvested water was recovered and weighed.

Throughout the AWH procedure, the sorbent's temperature variations were carefully monitored. The findings showed that when exposed to air, the composite's surface temperature rose from 24 °C (room temperature) to around 26.4 °C (Figure S24 (a)). As the material sorbed water, heat was generated during the process, which is what caused the temperature to rise. When not adequately controlled or employed in polymer dissolution, this energy slowly leaks

out into the environment, and no further variation in temperature is monitored (see section S2.15). As soon as the box was sealed, the BAGY's surface temperature rose by 20 °C, from 26 to 46 ° C, within the first 30 min of one-sun exposure, which caused water to desorb. After that, the water released at a significantly quicker pace between 30 min and 4 h, consuming a significant amount of heat and causing a slow shift in temperature. From this point on, the temperature stayed constant at 68 °C, showing that a balance between heat transfer and solar energy absorption had been reached. The heated sample quickly cooled to 32 °C in just 60 seconds after being shielded from the sun's radiation. It was proven through the experiment that BAGY-based AWH can generate water using 58.1% of the solar energy (see section S2.16). The sorption process is a passive process that can take many hours, but the desorption process is an active process that can be triggered by an external heat source; hence its rate tends to be greater. The time needed to achieve 81% of desorption was 210 min in a closed atmosphere with one-sun or irradiation. Due to the restricted water condensation rate in enclosed environment, the desorption equilibrium could be reached within 400 min (Figure 3 (a)). A total of 6.09 g of water was recovered, resulting in a total water recovery efficiency of 74%.

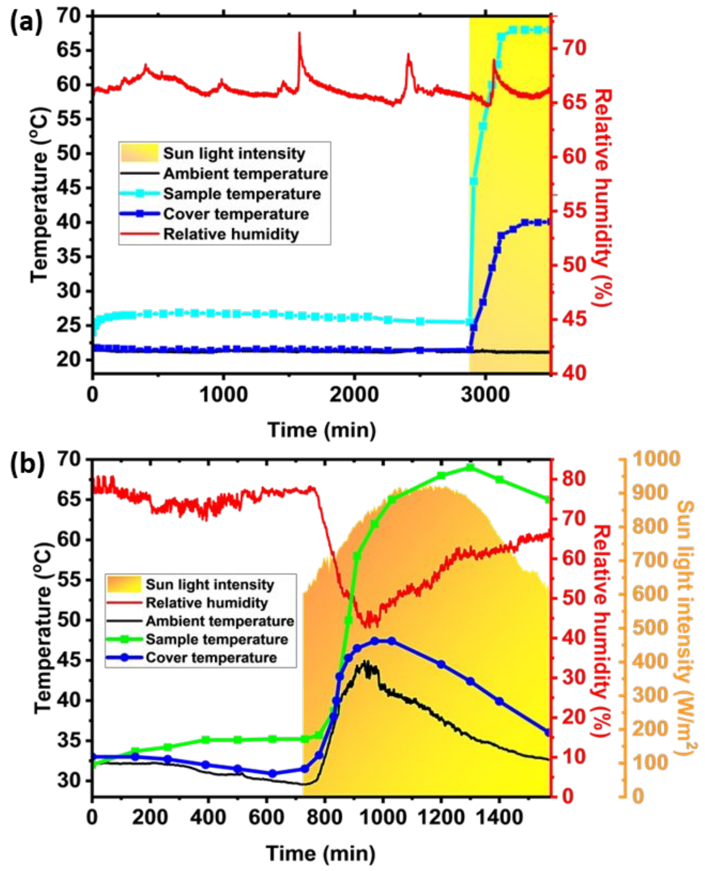

Figure S24 Temperature profile of ambient air, cover, sample, and ambient RH in (a) indoor and (b) outdoor AWH experiments.

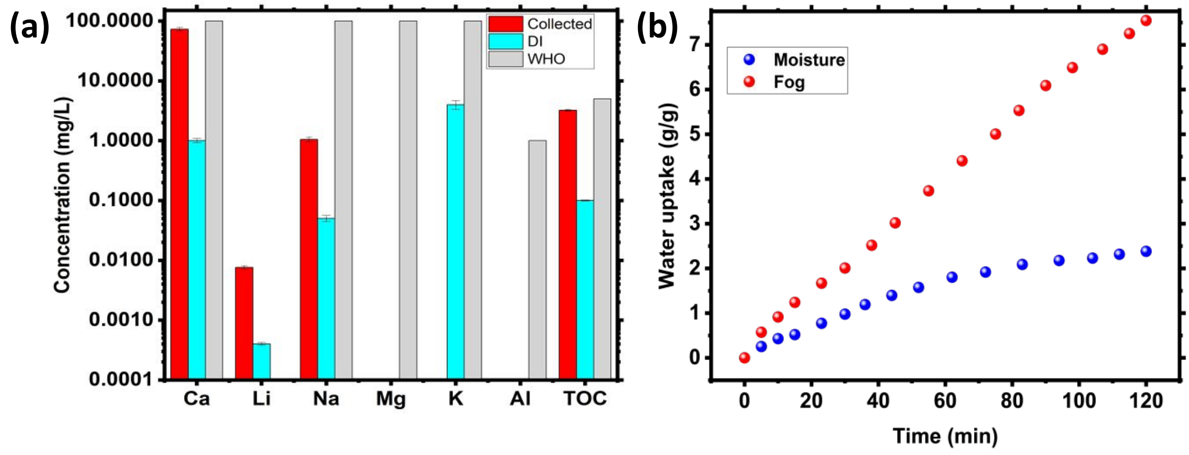

Figure S25 (a) Quality of water collected from outdoor tests (bulk water around samples) and (b) BAGY's water uptake behavior from fog and moisture (90%RH).

S2 Supplementary References

(1) Sun, Y.; Li, Y.; Chen, B.; Cui, M.; Xu, W.; Li, L. High-Efficiency Adsorption Performance of Cobalt Alginate / Graphene Oxide Aerogel Prepared by Green Method for Methylene Blue. *ChemistrySelect* **2022**, *202201216*. https://doi.org/10.1002/slct.202201216.

(2) Tabish, M. S.; Hanapi, N. S. M.; Wan Ibrahim, W. N.; Saim, N.; Yahaya, N. Alginate-Graphene Oxide Biocomposite Sorbent for Rapid and Selective Extraction of Non-Steroidal Anti-Inflammatory Drugs Using Micro-Solid Phase Extraction. *Indonesian Journal of Chemistry* **2019**, *19* (3), 684–695. https://doi.org/10.22146/ijc.38168.

(3) Das, M.; Aswathy, T. R.; Pal, S.; Naskar, K. Effect of Ionic Liquid Modified Graphene Oxide on Mechanical and Self-Healing Application of an Ionic Elastomer. *Eur Polym J* **2021**, *158*, 110691. https://doi.org/10.1016/J.EURPOLYMJ.2021.110691.

(4) Adel, M.; Ahmed, M. A.; Mohamed, A. A. A Facile and Rapid Removal of Cationic Dyes Using Hierarchically Porous Reduced Graphene Oxide Decorated with Manganese Ferrite. *FlatChem* **2021**, *26*. https://doi.org/10.1016/j.flatc.2021.100233.

(5) Silva, K. M. M. N.; Costa, B. L.; Nunes Dourado, L. F.; Silva, R. O.; Silva-Cunha, A.; Santos, A. K.; Resende, R. R.; Faria, P. E.; Campos Rubio, J. C.; Goulart, G. A. C.; Silva-Caldeira, P. P. Four Modified Sodium Alginate/Carboxymethylcellulose Blends for Prednisone Delivery. *J Appl Polym Sci* **2021**, *138* (19), 50383. https://doi.org/10.1002/app.50383.

(6) Hidayah, N. M. S.; Liu, W. W.; Lai, C. W.; Noriman, N. Z.; Khe, C. S.; Hashim, U.; Lee, H. C. Comparison on Graphite, Graphene Oxide and Reduced Graphene Oxide: Synthesis and Characterization. In *AIP Conference Proceedings*; AIP Publishing LLC AIP Publishing, 2017; Vol. 1892, p 150002. https://doi.org/10.1063/1.5005764.

(7) Fei, Y.; Li, Y.; Han, S.; Ma, J. Adsorptive Removal of Ciprofloxacin by Sodium Alginate/Graphene Oxide Composite Beads from Aqueous Solution. *J Colloid Interface Sci* **2016**, *484*, 196–204. https://doi.org/10.1016/j.jcis.2016.08.068.

(8) Gholizadeh, B. S.; Buazar, F.; Hosseini, S. M.; Mousavi, S. M. Enhanced Antibacterial Activity, Mechanical and Physical Properties of Alginate/Hydroxyapatite

Bionanocomposite Film. *Int J Biol Macromol* **2018**, *116*, 786–792. https://doi.org/10.1016/J.IJBIOMAC.2018.05.104.

(9) Devi, R.; Tapadia, K.; Maharana, T. Casting of Carbon Cloth Enrobed Polypyrrole Electrode for High Electrochemical Performances. *Heliyon* **2020**, *6* (1), e03122. https://doi.org/10.1016/j.heliyon.2019.e03122.

(10) Xu, Y.; Mu, H.; Han, X.; Sun, T.; Fan, X.; Lv, B.; Pan, Z.; Song, Y.; Song, C. A Simple, Flexible, and Porous Polypyrrole-Wax Gourd Evaporator with Excellent Light Absorption for Efficient Solar Steam Generation. *Int J Energy Res* **2021**, *45* (15), 21476–21486. https://doi.org/10.1002/er.7195.

(11) Sadeghnezhad, M.; Ghorbani, M.; Nikzad, M. Industrial Crops & Products Synthesis of Magnetic Polypyrrole Modified Sodium Alginate Nanocomposite with Excellent Antibacterial Properties and Optimization of Dye Removal Performance Using RSM. *Ind Crops Prod* **2022**, *186* (June), 115192. https://doi.org/10.1016/j.indcrop.2022.115192.

(12) Wu, M.; Ding, S.; Deng, L.; Wang, X. PPy Nanotubes-Enabled in-Situ Heating Nanofibrous Composite Membrane for Solar-Driven Membrane Distillation. *Sep Purif Technol* **2022**, *281* (July 2021), 119995. https://doi.org/10.1016/j.seppur.2021.119995.

(13) Luu, C. H.; Nguyen, G.; Le, T. T.; Nguyen, T. M. N.; Giang Phan, V. H.; Murugesan, M.; Mathiyalagan, R.; Jing, L.; Janarthanan, G.; Yang, D. C.; Li, Y.; Thambi, T. Graphene Oxide-Reinforced Alginate Hydrogel for Controlled Release of Local Anesthetics: Synthesis, Characterization, and Release Studies. *Gels* **2022**, *8* (4). https://doi.org/10.3390/gels8040246.

(14) Minh Dat, N.; Minh Huong, L.; Tien Dat, N.; Ba Thinh, D.; Ngoc Trinh, D.; Thi Huong Giang, N.; Thanh Phong, M.; Huu Hieu, N. Synthesis of Hygroscopic Sodium Alginate-Modified Graphene Oxide: Kinetic, Isotherm, and Thermodynamic Study. *Eur Polym J* **2022**, *174* (January), 111333. https://doi.org/10.1016/j.eurpolymj.2022.111333.

(15) Varghese, A. M.; Reddy, K. S. K.; Singh, S.; Karanikolos, G. N. Performance Enhancement of CO2 Capture Adsorbents by UV Treatment: The Case of Self-Supported Graphene Oxide Foam. *Chemical Engineering Journal* **2020**, *386* (October 2019), 124022. https://doi.org/10.1016/j.cej.2020.124022.

(16) Platero, E.; Fernandez, M. E.; Bonelli, P. R.; Cukierman, A. L. Graphene Oxide/Alginate Beads as Adsorbents: Influence of the Load and the Drying Method on Their Physicochemical-Mechanical Properties and Adsorptive Performance. *J Colloid Interface Sci* **2017**, *491*, 1–12. https://doi.org/10.1016/J.JCIS.2016.12.014.

(17) Lentz, L.; Mayer, D. A.; Dogenski, M.; Ferreira, S. R. S. Hybrid Aerogels of Sodium Alginate/Graphene Oxide as Efficient Adsorbents for Wastewater Treatment. *Mater Chem Phys* **2022**, *283* (January), 125981. https://doi.org/10.1016/j.matchemphys.2022.125981.

(18) Eltaweil, A. S.; Mamdouh, I. M.; Abd El-Monaem, E. M.; El-Subruiti, G. M. Highly Efficient Removal for Methylene Blue and Cu2+onto UiO-66 Metal-Organic Framework/Carboxylated Graphene Oxide-Incorporated Sodium Alginate Beads. *ACS Omega* **2021**. https://doi.org/10.1021/acsomega.1c03479.

(19) Sandu, A. E.; Nita, L. E.; Chiriac, A. P.; Pamfil, D.; Tudorachi, N.; Rusu, A. G. New Hydrogel Network Based on Alginate and a Spiroaceta Copolymer. *Gels* **2021**, *7* (241).

(20) Entezari, A.; Ejeian, M.; Wang, R. Super Atmospheric Water Harvesting Hydrogel with Alginate Chains Modified with Binary Salts. *ACS Mater Lett* **2020**, *2* (5), 471–477. https://doi.org/10.1021/acsmaterialslett.9b00315.

(21) Yumin, A.; Liguo, D.; Yi, Y.; Yongna, J. Mechanical Properties of an Interpenetrating Network Poly(Vinyl Alcohol)/Alginate Hydrogel with Hierarchical Fibrous Structures. *RSC Adv* **2022**, *12* (19), 11632–11639. https://doi.org/10.1039/d1ra07368k.

(22) Liu, Y.; Zhang, L.; Tang, Y.; Zhu, L. Study on the Preparation and Adsorption Properties of Sodium Alginate Graft Polyacrylic Acid/Graphite Oxide Composite Hydrogel. *Polymer Science - Series A* **2021**, *63* (2), 133–142. https://doi.org/10.1134/S0965545X21020061.

(23) Soleimanpour, M.; Mirhaji, S. S.; Jafari, S.; Derakhshankhah, H.; Mamashli, F.; Nedaei, H.; Karimi, M. R.; Motasadizadeh, H.; Fatahi, Y.; Ghasemi, A.; Nezamtaheri, M. S.; Khajezade, M.; Teimouri, M.; Goliaei, B.; Delattre, C.; Saboury, A. A. Designing a New Alginate-Fibrinogen Biomaterial Composite Hydrogel for Wound Healing. *Sci Rep* **2022**, *12* (1), 1–17. https://doi.org/10.1038/s41598-022-11282-w.

(24) Zhang, Z.; Lin, T.; Li, S.; Chen, X.; Que, X.; Sheng, L.; Hu, Y.; Peng, J.; Ma, H.; Li, J.; Zhang, W.; Zhai, M. Polyacrylamide/Copper-Alginate Double Network Hydrogel Electrolyte with Excellent Mechanical Properties and Strain-Sensitivity. *Macromol Biosci* **2022**, *22* (2). https://doi.org/10.1002/mabi.202100361.

(25) Thakur, S.; Verma, A.; Raizada, P.; Gunduz, O.; Janas, D.; Alsanie, W. F.; Scarpa, F.; Thakur, V. K. Bentonite-Based Sodium Alginate/ Dextrin Cross-Linked Poly (Acrylic Acid) Hydrogel Nanohybrids for Facile Removal of Paraquat Herbicide from Aqueous Solutions. *Chemosphere* **2022**, *291* (November), 133002. https://doi.org/10.1016/j.chemosphere.2021.133002.

(26) Askarieh, M.; Farshidi, H.; Rashidi, A.; Pourreza, A.; Alivand, M. S. Comparative Evaluation of MIL-101(Cr)/Calcium Alginate Composite Beads as Potential Adsorbents for Removing Water Vapor from Air. *Sep Purif Technol* **2022**, *291*, 120830. https://doi.org/10.1016/j.seppur.2022.120830.

(27) Ma, J.; Zhang, M.; Ji, M.; Zhang, L.; Qin, Z.; Zhang, Y.; Gao, L.; Jiao, T. Magnetic Graphene Oxide-Containing Chitosan-sodium Alginate Hydrogel Beads for Highly Efficient and Sustainable Removal of Cationic Dyes. *Int J Biol Macromol* **2021**, *193* (August), 2221–2231. https://doi.org/10.1016/j.ijbiomac.2021.11.054.

(28) Xu, J.; Li, T.; Chao, J.; Wu, S.; Yan, T.; Li, W.; Cao, B.; Wang, R. Efficient Solar-Driven Water Harvesting from Arid Air with Metal–Organic Frameworks Modified by Hygroscopic Salt. *Angewandte Chemie - International Edition* **2020**, *59* (13), 5202–5210. https://doi.org/10.1002/anie.201915170.

(29) Kim, S.; Liang, Y.; Kang, S.; Choi, H. Solar-Assisted Smart Nanofibrous Membranes for Atmospheric Water Harvesting. *Chemical Engineering Journal* **2021**, *425* (June), 131601. https://doi.org/10.1016/j.cej.2021.131601.

(30) Zhao, F.; Zhou, X.; Liu, Y.; Shi, Y.; Dai, Y.; Yu, G. Super Moisture-Absorbent Gels for All-Weather Atmospheric Water Harvesting. *Advanced Materials* **2019**, *31* (10). https://doi.org/10.1002/adma.201806446.

(31) Chen, X.; Yang, Y.; Guan, Y.; Luo, C.; Bao, M.; Li, Y. A Solar-Heated Antibacterial Sodium Alginate Aerogel for Highly Efficient Cleanup of Viscous Oil Spills. *J Colloid Interface Sci* **2022**, *621*, 241–253. https://doi.org/10.1016/j.jcis.2022.04.073.

(32) Ren, P.; Wei, D.; Liang, M.; Xu, L.; Zhang, T.; Zhang, Q. Alginate/Gelatin-Based Hybrid Hydrogels with Function of Injecting and Encapsulating Cells in Situ. *Int J Biol Macromol* **2022**, *212* (May), 67–84. https://doi.org/10.1016/j.ijbiomac.2022.05.058.

(33) Seike, M.; Asaumi, Y.; Kawashima, H.; Hirai, T.; Nakamura, Y.; Fujii, S. Morphological and Chemical Stabilities of Polypyrrole in Aqueous Media for 1 Year. *Polym J* **2022**, *54* (2), 169–178. https://doi.org/10.1038/s41428-021-00572-1.

(34) Luceño-Sánchez, J. A.; Díez-Pascual, A. M. Grafting of Polypyrrole-3-Carboxylic Acid to the Surface of Hexamethylene Diisocyanate-Functionalized Graphene Oxide. *Nanomaterials* **2019**, *9* (8), 1095. https://doi.org/10.3390/nano9081095.

(35) Vigmond, S. J.; Ghaemmaghami, V.; Thompson, M. Raman and Resonance-Raman Spectra of Polypyrrole with Application to Sensor – Gas Probe Interactions. *https://doi.org/10.1139/v95-209* **2011**, *73* (10), 1711–1718. https://doi.org/10.1139/V95-209.

(36) Yang, S.; Shen, C.; Liang, Y.; Tong, H.; He, W.; Shi, X.; Zhang, X.; Gao, H. J. Graphene Nanosheets-Polypyrrole Hybrid Material as a Highly Active Catalyst Support for Formic Acid Electro-Oxidation. *Nanoscale* **2011**, *3* (8), 3277–3284. https://doi.org/10.1039/c1nr10371g.

(37) Yao, H.; Zhang, P.; Huang, Y.; Cheng, H.; Li, C.; Qu, L. Highly Efficient Clean Water Production from Contaminated Air with a Wide Humidity Range. *Advanced Materials* **2020**, *32* (6), 1–8. https://doi.org/10.1002/adma.201905875.

(38) Yao, W.; Zhu, X.; Xu, Z.; Davis, R. A.; Liu, G.; Zhong, H.; Lin, X.; Dong, P.; Ye, M.; Shen, J. Loofah Sponge-Derived Hygroscopic Photothermal Absorber for All-Weather Atmospheric Water Harvesting. *ACS Appl Mater Interfaces* **2022**, *14* (3), 4680–4689. https://doi.org/10.1021/acsami.1c20576.

(39) Hou, Y.; Sheng, Z.; Fu, C.; Kong, J.; Zhang, X. Hygroscopic Holey Graphene Aerogel Fibers Enable Highly Efficient Moisture Capture, Heat Allocation and Microwave Absorption. *Nat Commun* **2022**, *13* (1), 1–12. https://doi.org/10.1038/s41467-022-28906-4.

(40) Mittal, H.; Al Alili, A.; Alhassan, S. M. Adsorption Isotherm and Kinetics of Water Vapors on Novel Superporous Hydrogel Composites. *Microporous and Mesoporous Materials* **2020**, *299*, 110106. https://doi.org/10.1016/J.MICROMESO.2020.110106.

(41) Halsey, G. Physical Adsorption on Non-Uniform Surfaces. *J Chem Phys* **1948**, *16* (10), 931–937. https://doi.org/10.1063/1.1746689.

(42) Toribio, F.; Bellat, J. P.; Nguyen, P. H.; Dupont, M. Adsorption of Water Vapor by Poly(Styrenesulfonic Acid), Sodium Salt: Isothermal and Isobaric Adsorption Equilibria. *J Colloid Interface Sci* **2004**, *280* (2), 315–321. https://doi.org/10.1016/j.jcis.2004.08.009.

(43) Calabrese, L.; Brancato, V.; Bonaccorsi, L.; Frazzica, A.; Caprì, A.; Freni, A.; Proverbio, E. Development and Characterization of Silane-Zeolite Adsorbent Coatings for Adsorption Heat Pump Applications. *Appl Therm Eng* **2017**, *116*, 364–371. https://doi.org/10.1016/j.applthermaleng.2017.01.112.

(44) Mittal, H.; al Alili, A.; Alhassan, S. M. Capturing Water Vapors from Atmospheric Air Using Superporous Gels. *Sci Rep* **2022**, *12* (1), 1–13. https://doi.org/10.1038/s41598-022-08191-3.

(45) Kim, H.; Cho, H. J.; Narayanan, S.; Yang, S.; Furukawa, H.; Schiffres, S.; Li, X.; Zhang, Y. B.; Jiang, J.; Yaghi, O. M.; Wang, E. N. Characterization of Adsorption Enthalpy of Novel Water-Stable Zeolites and Metal-Organic Frameworks. *Sci Rep* **2016**, *6*. https://doi.org/10.1038/srep19097.
